# Affecting *Rhomboid-3* Function Causes a Dilated Heart in Adult *Drosophila*

**DOI:** 10.1371/journal.pgen.1000969

**Published:** 2010-05-27

**Authors:** Lin Yu, Teresa Lee, Na Lin, Matthew J. Wolf

**Affiliations:** 1 Division of Cardiology, Department of Medicine, Duke University Medical Center, Durham, North Carolina, United States of America; 2 Department of Cell Biology, Duke University, Durham, North Carolina, United States of America; 3 Institute of Molecular Medicine, Peking University, Beijing, China; University of California San Francisco, United States of America

## Abstract

*Drosophila* is a well recognized model of several human diseases, and recent investigations have demonstrated that *Drosophila* can be used as a model of human heart failure. Previously, we described that optical coherence tomography (OCT) can be used to rapidly examine the cardiac function in adult, awake flies. This technique provides images that are similar to echocardiography in humans, and therefore we postulated that this approach could be combined with the vast resources that are available in the fly community to identify new mutants that have abnormal heart function, a hallmark of certain cardiovascular diseases. Using OCT to examine the cardiac function in adult *Drosophila* from a set of molecularly-defined genomic deficiencies from the DrosDel and Exelixis collections, we identified an abnormally enlarged cardiac chamber in a series of deficiency mutants spanning the rhomboid 3 locus. Rhomboid 3 is a member of a highly conserved family of intramembrane serine proteases and processes Spitz, an epidermal growth factor (EGF)–like ligand. Using multiple approaches based on the examination of deficiency stocks, a series of mutants in the rhomboid-Spitz–EGF receptor pathway, and cardiac-specific transgenic rescue or dominant-negative repression of EGFR, we demonstrate that rhomboid 3 mediated activation of the EGF receptor pathway is necessary for proper adult cardiac function. The importance of EGF receptor signaling in the adult *Drosophila* heart underscores the concept that evolutionarily conserved signaling mechanisms are required to maintain normal myocardial function. Interestingly, prior work showing the inhibition of ErbB2, a member of the EGF receptor family, in transgenic knock-out mice or individuals that received herceptin chemotherapy is associated with the development of dilated cardiomyopathy. Our results, in conjunction with the demonstration that altered ErbB2 signaling underlies certain forms of mammalian cardiomyopathy, suggest that an evolutionarily conserved signaling mechanism may be necessary to maintain post-developmental cardiac function.

## Author Summary

Heart failure is a common cardiovascular disease that is characterized by problems with the ability of the heart muscle to contract, called impaired systolic function, and an enlarged heart chamber. Model systems of human heart failure are necessary to facilitate the screening and identification of genes and genetic variations that either cause or influence the development and progression of this disease. To better understand these genes, we conducted a genetic screen employing molecularly defined deletions throughout the genome of the adult fruit fly, *Drosophila melanogaster*. We used an optical coherence tomography imaging technique that provided images similar to echocardiography in humans to measure the cardiac function in adult flies. We identified mutants in members of the rhomboid protease family and epidermal growth factor receptor that cause an enlarged cardiac chamber. Interestingly, abnormalities in the function of members of the epidermal growth factor receptor family in humans that undergo certain chemotherapies are associated with the development of dilated cardiomyopathy and heart failure. Our results suggest that epidermal growth factor receptor signaling may be an evolutionarily conserved pathway that is necessary to maintain normal adult cardiac function.

## Introduction

The identification of genes that cause or modify cardiac dysfunction is required to understand the complex biology that is responsible for cardiomyopathies and heart failure in humans. The screening and identification of genetic mutants that affect cardiac function are facilitated by model systems of human heart failure. *Drosophila* has been used as a model of several human diseases and recent investigations have demonstrated that *Drosophila* can be used as a model of human heart failure, defined as an abnormally enlarged cardiac chamber when the heart is fully relaxed at end-diastole and an impaired systolic function [1]–[5]. Previously, we described that optical coherence tomography (OCT) can be used to examine the cardiac function in adult, awake flies [5]. OCT is a non-destructive, non-invasive imaging modality based on reflectivity of near-infrared light and provides detailed functional imaging of the adult fly heart in a manner similar to echocardiography in humans.

To identify new gene mutations that cause an enlarged cardiac chamber, we employed OCT to examine the cardiac chamber size in adult *Drosophila* from a set of the DrosDel and Exelixis collections that have molecularly-defined genomic deficiencies [6], [7]. During the course of our genetic screen, we identified abnormalities in cardiac chamber dimensions in a series of deficiency mutants spanning the rhomboid 3 locus. *Drosophila rhomboid 3* (*rho3*), also known as *roughoid*/*ru*, is a member of a highly conserved family of intramembrane serine proteases and processes Spitz, an epidermal growth factor (EGF)-like ligand [8]–[14]. Initially, isolated in a genetic screen of embryonic developmental defects, rhomboids are essential for proper EGF receptor (EGFR) signaling in *Drosophila*. In fact, rhomboid and EGFR signaling is necessary for the development of the fly eye, trachea, and embryonic somatic musculature and many other places in the fly [7], [8], [15]–[17].

Spitz and EGFR signaling are required for the specification and diversification of *Drosophila* embryonic muscle progenitors [16]. During development, individual muscle groups are differentially sensitive to the level of EGFR signaling that results from the spatial restriction of Spitz and other ligands [14]. Rhomboids have been implicated in this process and rhomboid-1 (*rho*) has been shown to be required for dorsal acute 1 (DA1) muscle formation in the embryo [16].

The embryonic dorsal vessel that becomes the adult fly heart develops through an orchestrated series of spatially restricted mesodermal and ectodermal signals involving dpp, Wnt, and hedgehog [18]–[22]. During morphogenesis the adult heart, also known as the conical chamber, arises from the embryonic aorta while the embryonic dorsal vessel degenerates to become the terminal chamber of the adult circulatory system [23], [24]. Recently, Perrin *et al.* have begun to elucidate the signaling pathways in the transition from the fly embryonic to adult heart through an examination of gene profiling studies [25]. This work has identified roles for FGF, Wnt, and PDGF-VEGF signaling in the morphogenesis of the adult fly heart. However, the signaling pathways that are necessary for maintenance of cardiac function after establishment of the adult *Drosophila* circulatory system are only recently beginning to be identified [2], [26].

Our results demonstrate that a partial inhibition of rho3 causes an enlargement in the cardiac chamber in adult *Drosophila* through inhibition of the EGFR pathway. We show that the mutant *roughoid-1*, designated *ru^1^*, encodes a missense mutation in rho3 corresponding to a premature stop codon and has a recessive cardiac phenotype. While the *ru^1^* homozygote embryonic dorsal vessel and pupal heart are not significantly different from *w^1118^*, the adult heart in *ru^1^* homozygote is larger than *w^1118^*, consistent with the functional measurements obtained by OCT. Furthermore, the restoration of *rho3* by cardiac expression of transgenic wild-type *rho3* rescues cardiac function in the context of genomic deficiencies for endogenous *rho3* or the *ru^1^* mutant.

Since rho3 is important in processing of Spitz and subsequent EGFR signaling, we examined the effects of alteration in Spitz and EGFR on cardiac function in adult *Drosophila*. Cardiac specific expression of a processed form of activated Spitz rescued the abnormality in cardiac phenotype that was observed in *rho3* mutants. Additionally, expression of EGFR also restores cardiac function in the context of genomic deficiencies in *rho3* or the *ru^1^* mutant. Finally, a temperature-sensitive, cardiac specific expression of dominant-negative EGFR in adult *Drosophila* causes a progressive enlargement of the cardiac chamber that is observed by serial OCT measurements. Collectively, these results suggest that deficiencies in *rho3* and its downstream components in the EGFR signaling pathway lead to an enlargement of the cardiac chamber in adult *Drosophila*.

## Results

### Identification of a genomic deficiency on chromosome 3L that is associated with an enlarged cardiac chamber

**Figure 1:**
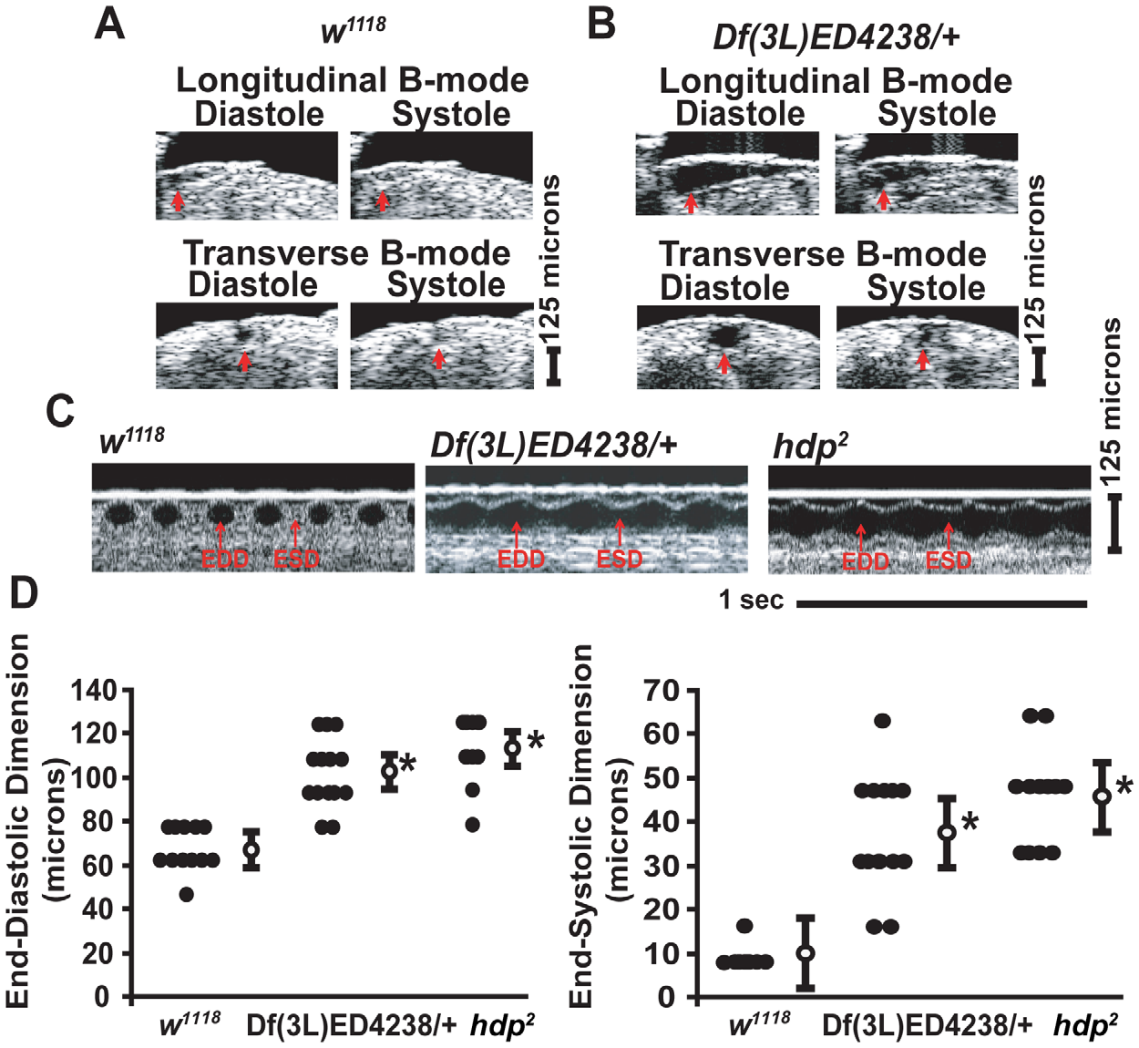
Identification of a genomic deletion mutant that has an enlarged cardiac chamber. (A,B) Representative B-mode optical coherence tomography (OCT) images of *w^1118^* (A) and *Df(3L)ED4238*/+ (B) during diastole and systole in longitudinal and transverse orientations. The red arrows denote the cardiac chamber. A 125 micron standard is shown. (C) Representative m-mode OCT images of *w^1118^*, *Df(3L)ED4238*/+, and *hdp^2^*. The position of end-diastolic dimension (EDD) and end-systolic dimension (ESD) during the cardiac cycle are indicated by red arrows. Each m-mode represents one second and a 125 micron standard is shown. (D) Summary data of cardiac measurements for end-diastolic dimension (EDD) and end-systolic dimension (ESD) from *w^1118^*, *Df(3L)ED4238*/+, and *hdp^2^*. Closed circles represent individual measurements for each group, open circles represent the mean ± SEM for each group. n = 13−17 flies per group. *p<0.05 for the indicated measurements compared to *w^1118^* using an ANOVA with Bonferroni correction for multiple comparisons for EDD or Kruskal-Wallis test with a Dunn's test for multiple comparisons for ESD.

**Figure 2:**
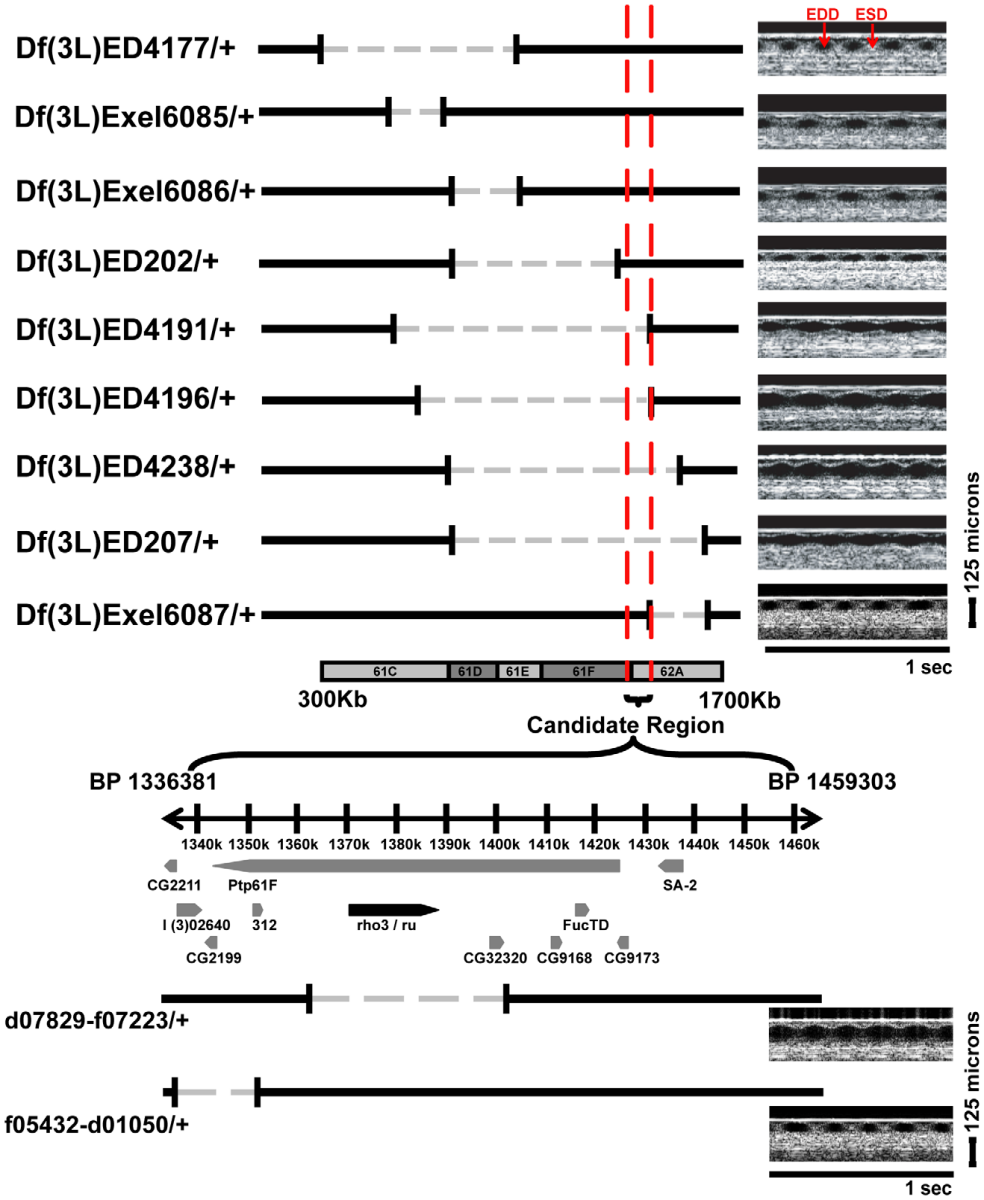
Schematic representation of the candidate region of chromosome 3L. Each line represents the region of the genome that is deleted in gray with the DrosDel or Exelixis stock designation to the left of the line. Representative OCT m-modes corresponding to each strain are shown to the right. A 125 micron standard is shown. Inclusion of regions that correspond to dilated cardiac phenotype and exclusion of regions that had normal cardiac function is represented by the red lines and narrows the interval to ∼122 kb of genomic DNA. The genes encoded within the candidate region are shown below with *ru* (*rho3*) represented in black. The *d07829-f07223*/+ and *f05432-d01050*/+ custom genomic deletions with representative OCT m-modes are shown below.

As part of a systematic, genome wide screen in *Drosophila* to identify candidate gene mutations that cause abnormalities in adult cardiac function, we employed optical coherence tomography to examine the cardiac function in awake, adult *Drosophila* [5]. We examined flies with molecularly-defined genomic deficiencies along the 3L chromosome from the DrosDel and Exelixis collections and identified a set of mutants that had a dilated heart manifest as enlarged end-diastolic dimension (EDD) and end-systolic dimension (ESD). We also calculated fractional shortening (FS), a parameter that correlates with contractile function and has been used to describe the cardiac function in a variety of models of cardiovascular disease [27]–[32] (Figure S1). All deficiency mutants were bred into a *w^1118^* background to remove possible influences that balancer chromosomes may have on the cardiac phenotype. Therefore, all the genomic deficiency mutants were examined as heterozygotes. In an initial screen of 32 deficiency stocks (Table S1), we identified a markedly enlarged cardiac chamber in mutant *Df(3L)ED4238*/*+* (Figure 1A–1D). The severity of the cardiac chamber enlargement observed in *Df(3L)ED4238*/*+* was indistinguishable from the cardiac dysfunction in the troponin-I mutant, *hdp^2^*, that has been previously well-characterized [5]. An examination of the cardiac chamber size in deficiency mutants that surrounded the region corresponding to *Df(3L)ED4238*/*+* revealed three additional genomic deficiencies, *Df(3L)ED4196*/+, *Df(3L)ED207*/+, and *Df(3L)ED4191*/+, that had abnormal cardiac dimensions thereby narrowing the initial genomic interval from ∼800Kb to a candidate region encompassing ∼120 Kb that encoded 9 genes (Figure 2 and Table 1). Since the resolution of OCT is limited to 8 microns, we also examined the data in a dichotomized manner. We defined “normal” diastolic cardiac size as an EDD <90 microns and an “enlarged heart” as an EDD > 90 microns. Additionally, “normal” systolic cardiac dimension was defined as <20 microns and “abnormal” systolic dimension as > 20 microns. The examination of dichotomized data also supported the observation that *Df(3L)4238*/+, *Df(3L)ED4196*/+, *Df(3L)ED207*/+, and *Df(3L)ED4191*/+ had abnormal cardiac chamber sizes.

**Table 1:** Summary of cardiac parameters of adult *Drosophila* that have genomic deficiencies along 3L.

	N	EDD ± SEM (microns)	ESD ± SEM (microns)	FS ± SEM (%)	Dilated (EDD>90 microns)	Impaired systolic function (ESD>20 microns)
*W^1118^*	17	66±8	<10	>85	N	N
*Df(3L)ED4177*/+	8	83±6	<10	>88	N	N
*Df(3L)ED202*/+	10	75±2	<10	>87	N	N
*Df(3L)Exel6085*/+	12	65±5	<10	>85	N	N
*Df(3L)Exel6086*/+	8	78±5	<10	>88	N	N
*Df(3L)ED4191*/+	8	106±4*	43±3*	59±3	Y	Y
*Df(3L)ED4196*/+	10	121±4*	55±5*	55±3	Y	Y
*Df(3L)ED4238*/+	13	103±4*	37±4*	64±2	Y	Y
*Df(3L)ED207*/+	17	109±4*	49±3*	55±3	Y	Y
*Df(3L)Exel6087*/+	12	77±5	<10	>87	N	N
*f05423-d04958*/+	8	102±7*	27±6*	73±3	Y	Y
*d07829-f07223*/+	15	105±9*	35±7*	68±5	Y	Y
*f05423-d01050*/+	16	81±4	<10	>89	N	N
*Mi{ET1}CG32320^MB04181^*/+	10	80±10	<10	>88	N	N
*Mi{ET1}CG32320^MB08823^*/+	9	80±10	<10	>88	N	N
*hdp^2^*	12	109±7*	43±5*	61±3	Y	Y

The data represent the cardiac measurements in adult flies obtained by OCT for end-diastolic dimension (EDD) in microns, end-systolic dimension (ESD) in microns, and fractional shortening (FS). FS was calculated as (EDD-ESD)/EDD ×100. Values are expressed as the mean +/- SE. N represents the number of samples per group. *p<0.05 for EDD compared to *w^1118^* using ANOVA with Bonferroni correction for multiple comparisons or ESD compared to *w^1118^* using Kruskal-Wallace with Dunn's correction for multiple comparisons. Additionally, the data is represented in a binary manner with “Dilated” defined as an EDD > 90 microns and “Impaired Systolic Function” defined as an ESD >20 microns where “N”  =  no and “Y” = yes.

**Figure 3:**
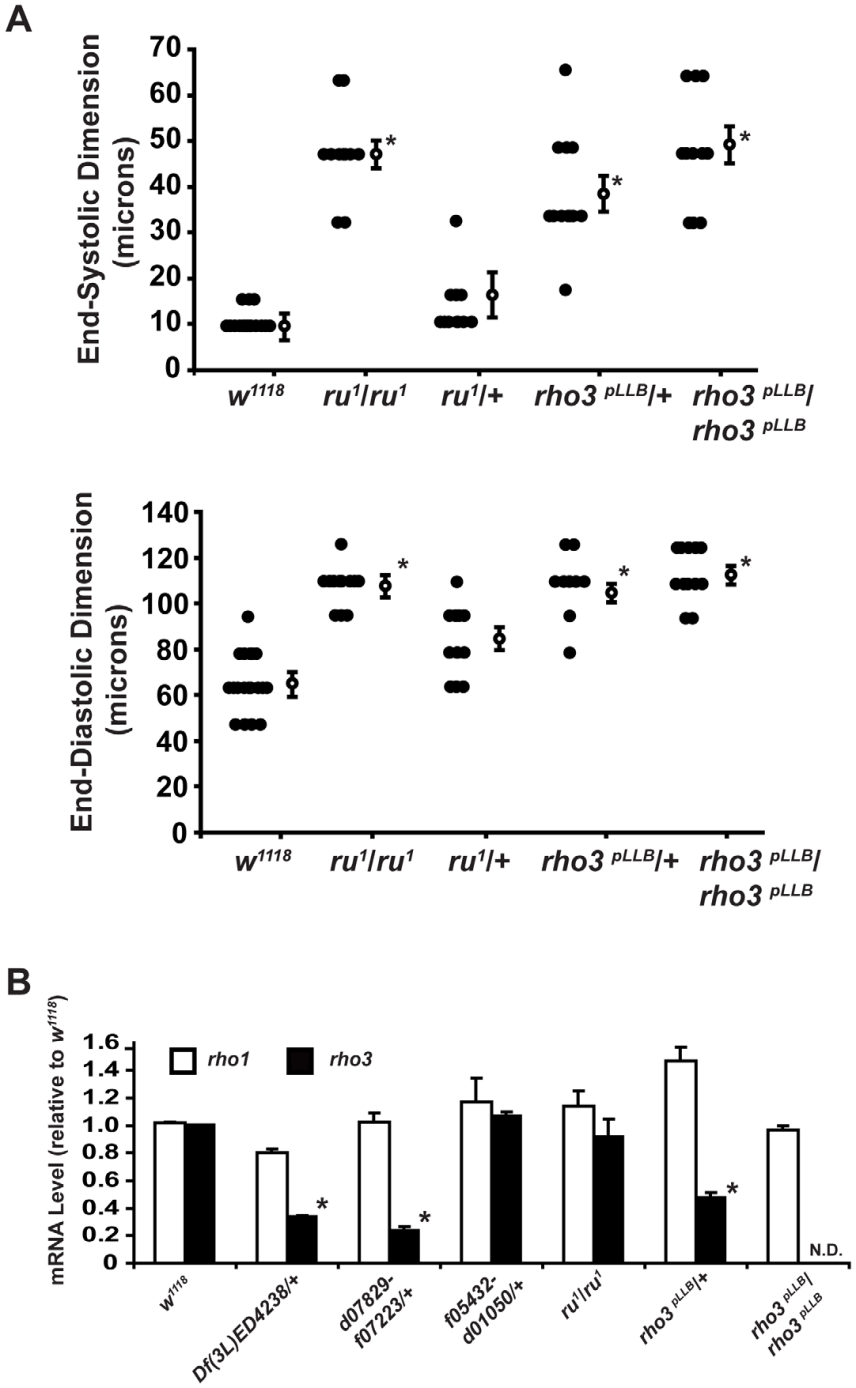
Flies with mutant alleles encoding *ru^1^* or *rho3^pLLB^* have enlarged cardiac chambers. (A) Summary data of cardiac measurements for end-systolic dimension (ESD) (top) and end-diastolic dimension (EDD) (bottom) from *w^1118^*, *ru^1^* homozygotes (designated *ru^1^/ru^1^*), *ru^1^* heterozygotes (*ru^1^*/+), *rho3^pLLB^* heterozygotes (*rho3^pLLB^/+*), and *rho3^pLLB^* homozygotes (*rho3^pLLB^/rho3^pLLB^*). Closed circles represent individual measurements for each group, open circles represent the mean ± SEM for each group. n = 8−17 flies per group. *p<0.05 for the indicated measurements by ANOVA with Bonferroni correction for multiple comparisons for EDD or Kruskal-Wallis test with a Dunn's test for multiple comparisons for ESD. (B) Summary data for expression of mRNA for *rho1* or *rho3* in *w^1118^*, *Df(3L)ED4238/+*, heterozygous genomic deficiencies *d07829-f07223*/+ or *f05432-d01050*/+, *ru^1^* homozygous mutants (*ru^1^/ru^1^*), *rho3^pLLB^* heterozygotes (*rho3^pLLB^/+*), or *rho3^pLLB^* homozygotes (*rho3^pLLB^/rho3^pLLB^*). Values are expressed as the mean mRNA expression +/- SEM relative to *w^1118^* and represent three independent experiments using 10 flies per group per experiment. Rho3 mRNA was not detected (N.D.) in *rho3^pLLB^* homozygotes. *p<0.05 for *rho3* in *Df(3L)ED4328/+*, *d07829-f07223*/+, or *rho3^pLLB^/+* versus *w^1118^* by t-test.

To identify the candidate gene that was responsible for the abnormal cardiac phenotype within the region, mutants from the Exelixis P-element insertion collection were used to engineer mutant *Drosophila* that had molecularly-defined deficiencies spanning the genomic region of interest [6]. The deficiency mutant, *f05423-d04958*/+, spanned the genomic region that was inferred from the initial screen and had an enlarged cardiac chamber compared to *w^1118^* (EDD 102 +/- 7 microns and ESD 27 +/- 6 microns for *f05423-d04958*/+ versus EDD of 66 +/- 8 microns, and ESD of <10 microns for *w^1118^*) (Table 1). Interestingly, a smaller genomic deficiency, designated *d07829-f07223*/+, that encompassed the gene encoding rhomboid 3, (herein referred to as *rho3*) and *CG32320* also demonstrated an enlarged cardiac chamber (EDD 105 +/- 9 microns and ESD 35 +/- 7 microns for *d07829-f07223*/+) while a genomic deficiency, f05432-d01050/+, encoding several genes upstream of *rho3* had normal cardiac function (Figure 2 and Table 1). *rho3* mRNA levels were significantly decreased (∼25% in *d07829-f07223*/+ compared to *w^1118^*), while no different in *f05432-d01050*/+ mutant that has a genomic deficiency outside the *rho3* locus (Figure 3B). Furthermore, we observed normal cardiac function in the mutant *Df(3L)Exel6087*/+ that was heterozygous for a deficiency across the *rho1* and *rho2* loci but did not disrupt *rho3* (Table 1). Since the candidate region also contained the predicted gene *CG32320*, we examine the cardiac phenotype in two mutants that had the insertion of P-elements into the *GC32320* genomic regions. Mi{Et1}CG32320^MB04181^, inserted into the coding region of the 4th predicted exon of *CG32320*, and Mi{ET1}CG32320^MB08823^, inserted in the 5′ UTR region of *CG32320*, had normal cardiac phenotypes (Table 1). Based on these observations, we focused our attention on *rho3* as the candidate gene that was responsible for the abnormal cardiac phenotype observed in the deficiency mutants.

### The mutant *ru^1^* encodes a missense mutation in *rho3* and has an enlarged cardiac chamber

Next, we examined available mutants that spanned the candidate region and identified a cardiac abnormality in *roughoid (ru^1^)*. *ru^1^* was initially isolated by Sturtevant in 1918, has been shown to be a hypomorphic allele that results in a rough eye phenotype, and was subsequently mapped to the *rho3* locus [13], [33]. The *ru^1^* mutant had a C to T mutation at cDNA nucleotide position 163 that predicted a premature stop codon in *rho3* at amino acid position 55 (Figure S2); however, expression of recombinant Flag-tag fusion protein from cDNA isolated from *ru^1^* flies did not encode a truncated protein in S2 cells (Figure S2). Only homozygote *ru^1^* mutant flies had enlarged cardiac phenotype (EDD 107 +/− 3 microns and ESD 47 +/− 2microns (n = 12) for homozygote *ru^1^* mutants versus EDD 81 +/− 5 microns and ESD <10 microns (n = 12) for heterozygote *ru^1^* mutants) (Figure 3A). The abnormal recessive cardiac phenotype persisted after several rounds of backcrossing into the *w^1118^* genetic background (data not shown).

Interestingly, the initial identification of an enlarged cardiac chamber was made in heterozygous genomic deficiency mutants; however, abnormalities in cardiac chamber size were only found in homozygous *ru^1^* mutants. This observation is consistent with prior studies that identified *ru1* as a hypomorphic allele based on abnormalities in fly eye phenotypes [13]. We also examined the cardiac chamber size in *rho-3^PLLb^*, a mutant that has a break in the first intron of *rhomboid-3* and removes a portion of the *rhomboid-3* coding sequence [13]. The *rho-3^PLLb^* allele has been described as a null allele, and therefore should lead, when heterozygous, to the same phenotype as the heterozygous genomic deficiencies *Df(3L)ED4328*/+. We used QRT-PCR to confirm that heterozygotes for *rho-3^PLLb^* had a 50% reduction in *rho3* mRNA, similar to *rho3* mRNA levels in heterozygous *Df(3L)ED4238*/+, while homozygotes for *rho-3^PLLb^* had non-detectable levels of *rho3* mRNA (Figure 3B). Mutants homozygous or heterozygous for the *rho-3^PLLb^* allele had enlarged cardiac chambers (Figure 3A and Table 2). The ESD in mutants for homozygous *rho-3^PLLb^* was slightly larger than that for heterozygous *rho-3^PLLb^* but did not achieve statistical significance.

**Table 2:** Summary of cardiac parameters of adult *Drosophila* with mutations in Rhomboids, Spitz, or Keren.

	N	EDD ± SEM (microns)	ESD ± SEM (microns)	FS ± SEM (%)	Dilated (EDD>90 microns)	Impaired systolic function (ESD>20 microns)
*ru^1^/+; rho^7M43^/+*	12	77±3	<10	>87	N	N
*rho3^pLLB^*/*rho3^pLLB^*	12	113±4	46±3	59±3	Y	Y
*rho3^pLLB^*/*+*	12	105±3	38±4	65±3	Y	Y
*rho 4-*Δ ˜/*rho 4-*Δ	11	75±3	<10	>87	N	N
*rho 6 null*/*rho 6 null*	7	78±3	<10	>87	N	N
*rho 4-*Δ/*rho 4-*Δ*; rho 6 null*/*rho 6 null*	11	81±4	<10	>88	N	N
*spi^1^*/+	9	82±3	<10	>88	N	N
*spi^s3547^*/+	10	78±5	<10	>87	N	N
*spi-SPC2*/*spi-SPC2*	11	114±4	36±5	67±4	Y	Y
*Keren^exc27-7-B^*/*Keren^exc27-7-B^*	11	73±3	<10	>86	N	N

The data represent the cardiac measurements in adult flies obtained by OCT for end-diastolic dimension (EDD) in microns, end-systolic dimension (ESD) in microns, and fractional shortening (FS). FS was calculated as (EDD-ESD)/EDD ×100. Values are expressed as the mean +/- SE. N represents the number of samples per group. Additionally, the data is represented in a binary manner with “Dilated” defined as an EDD >90 microns and “Impaired Systolic Function” defined as an ESD >20 microns where “N”  =  no and “Y” = yes.

Additionally, we examined the contribution of rho4 and rho6 abnormalities on cardiac function using mutants that were deficient for rho4, rho6 or both rho4 and rho6 (Freeman, M. unpublished data). Mutants that lacked rho4, rho6 or both rho4 and rho6 had normal cardiac function (Table 2). We also examined the potential contribution of rho1 and rho3 by examining the cardiac function in *ru^1^*/+, *rho^7M43^*/+ flies that were heterozygous for the *rho3*/*ru* allele and an amorphic allele of *rho1*. The *ru^1^*/+, *rho^7M43^*/+ mutants had normal cardiac function (Table 2), suggesting that cardiac abnormalities observed in the homozygous *ru^1^* mutants were not phenocopied by a combination of defects in rho3 and rho1. These data suggested that rho-3 inactivation is responsible for the observed enlarged heart phenotype; however, we could not definitely rule out the contribution from rho-1.

### The enlarged cardiac chamber in mutant *ru^1^* does not result from a developmental defect

Rhomboid-3 is expressed in embryos, pupae, and adult tissues including the heart (Table S2). Since alterations in embryonic dorsal vessel development can lead to abnormal cardiac phenotypes in adult *Drosophila*, the dorsal vessel morphology was evaluated in *Drosophila* embryos from stage 13 to 16. Transgenic *Drosophila* that express tinC-GFP in the context of *w^1118^* or *ru^1^* homozygotes demonstrated similar dorsal vessel morphology (data not shown). The heart in transgenic *Drosophila* that expressed tinC-GFP in the context of *w^1118^* or *ru^1^* homozygotes did not show a significant difference in overall morphology at different stages of pupal development (Figure S3).

Since adult homozygous *ru^1^* mutants had an enlarged cardiac chamber by OCT, the morphology of the adult cardiac chamber in *ru^1^* homozygous mutants was evaluated by histological sectioning. A comparison between *w^1118^* and *ru^1^* homozygous mutants was performed by examining 8 micron serial sections in the posterior portion of the A1 segment (see details in Materials and Methods section). Although the cardiac chamber size in *ru^1^* was larger than *w^1118^*, consistent with OCT results, and the cardiac chamber wall thickness in *ru^1^* homozygote mutants appeared thinner compared to *w^1118^*, the extra cardiac structures appeared similar between *ru^1^* and *w^1118^* (Figure S4).

**Figure 4:**
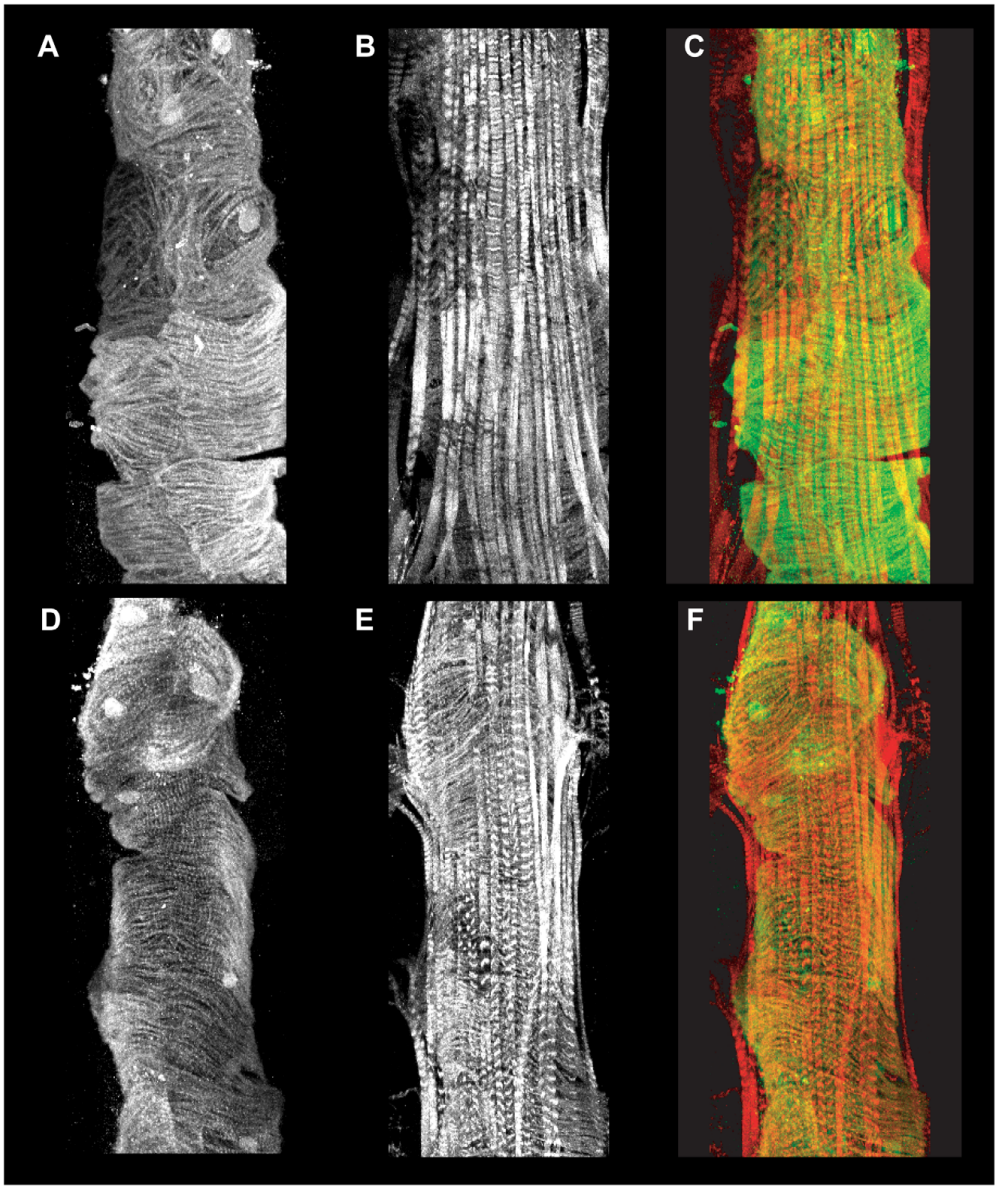
Evaluation of the A2 cardiac segment in homozygous *ru^1^* mutants. Panels are representative Z-stack confocal microscopy images of the A2 segment of adult hearts from *w^1118^* (A-C) and homozygous *ru^1^* mutants (D-F). Antibody staining of GFP (A and D) shows tinC positive cardiomyocytes and nuclei in monochrome. Texas-red phalloidin staining (B and E) shows actin structures in the cardiomyocytes and the extra cardiac (non-tinC positive) dorsal diaphragm muscle in monochrome. Merged imaged (C and F) show overlays of GFP (green) and actin (red) staining.

Moreover, the actin fibers in the dorsal diaphragm muscle, also known as the ventral longitudinal fibers, and cardiomyocytes in adult *Drosophila* hearts were examined via using confocal microscopy. Visualization of actin structures by staining with phalloidin demonstrated that the dorsal diaphragm muscle was similar between *ru^1^* homozygotes and *w^1118^*. The GFP positive cardiomyocyte nuclei and actin fiber organization were also similar (Figure 4). Overall, the tissue surrounding the adult hearts in dissected specimens was similar in *ru^1^* homozygotes and *w^1118^*. The functional abnormalities observed in the *ru^1^* homozygous mutants did not appear to be related to alterations in extra cardiac structures.

In addition, the other cardiac parameters, such as arrhythmia and heart rate, were not significantly different between *ru^1^* homozygotes and *w^1118^* in intact adults or in dissected adult hearts perfused with artificial hemolymph (Figure S5).

### Cardiac expression of Rho3, Spitz, or EGFR rescues the abnormal cardiac phenotype in *DF(3L)4238*/+ and homozygous *ru^1^* mutants

**Figure 5:**
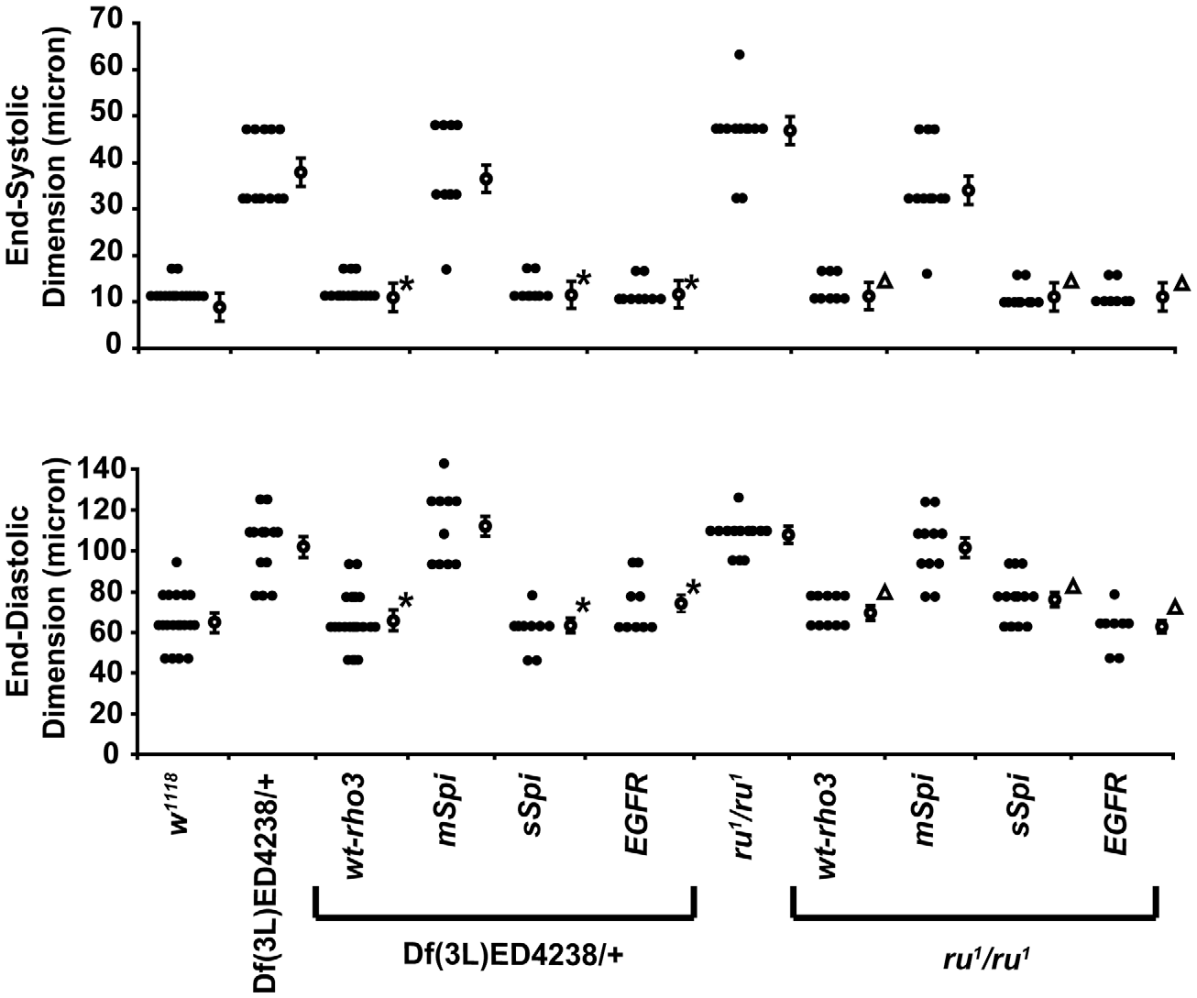
Cardiac expression of wt-rho3, soluble Spitz, or EGFR rescues the abnormal cardiac phenotype in *Df(3L)ED4238*/+ or homozygous *ru^1^* mutants. Summary data of cardiac measurements for end-systolic dimension (ESD) (top) and end-diastolic dimension (EDD) (bottom) from *w^1118^*, homozygous *ru^1^* mutants (*ru^1^*/*ru^1^*), *Df(3L)ED4238*/+, and transgenic flies harboring the driver *tinC*-Gal4 and UAS-wt-rho3, UAS-*mSpi,* UAS-*sSpi, or* UAS-*EGFR* in the context of the *Df(3L)ED4238*/+ or the homozygous *ru^1^* mutant. Closed circles represent individual measurements for each group, open circles represent the mean ± SEM for each group. n = 8−17 flies per group. n = 8−17 flies per group. *p<0.05 for the indicated measurements comparing *Df(3L)ED4238*/+ alone versus *Df(3L)ED4238*/+ expressing the indicated transgenes. Δp<0.05 for the indicated measurements comparing homozygous *ru^1^* alone versus homozygous *ru^1^* in the context of the indicated transgene. EDD measurements were analyzed by student's t-test and ESD measurements were analyzed by Mann-Whitney tests.

To confirm whether *rho3* was responsible for the abnormal cardiac phenotype, we engineered transgenic *Drosophila* that harbored wild-type (wt)-*rho3* under the UAS-promoter. In combination with cardiomyocyte specific tinC-Gal4 driver, we examined the effects of cardiac-specific expression of wt*-rho3* in the context of *Df(3L)ED4238*/+ or *ru^1^* homozygotes. The tinC-mediated expression of wt-*rho3* rescued the abnormal enlarged cardiac phenotype (Figure 5). Furthermore, the enlarged cardiac chamber phenotype that was observed in other genomic deficiencies spanning *rho3* [*Df(3L)ED4191*/+ or *Df(3L)ED4196*/+] was also rescued by wt-*rho3* (data not shown).

Since rho3 is known to process an inactive membrane-bound Spitz (mSpi) to an active soluble Spitz (sSpi) that can subsequently interact with EGFR, we examined the effects of these components on cardiac function in flies that had enlarged cardiac chambers attributed to genomic deficiencies [10]. The cardiac expression of sSpi, but not mSpi, rescued the abnormal cardiac phenotype observed in the *Df(3L)ED4238*/+ mutant (Figure 5). Furthermore, the cardiac-specific expression of EGFR in the context *Df(3L)ED4238*/+ also restored normal cardiac function. We also tried to examine the ability of constitutively active forms of EGFR to rescue the abnormal cardiac phenotype observed in the genomic deficiency mutants [34], [35]. The cardiac-specific expression of *EGFR^Act(2)^* resulted in a failure of flies to eclose from the late pupal state (data not shown).

Next, we examined the effects of cardiac-specific expression of *mSpi*, *sSpi*, and *EGFR* in the context of *ru^1^* homozygous mutant [10], [36], [37]. The cardiac-specific expression of sSpi or EGFR by tinC-Gal4 in the context of *ru^1^* homozygotes restored normal cardiac function while expression of mSpi by tinC-Gal4 in the context of *ru^1^* homozygotes still had an enlarged cardiac chamber (Figure 5).

Additionally, we examined *spi^1^*, a loss of function allele, and *spi^s3547^*, a P-element insertion mutant. Both mutants are lethal as homozygotes and therefore were examined in the heterozygous state [38]. *spi^1^*/+ and *spi^s3547^*/+ had normal cardiac function (Table 2). Interestingly, *spi-SCP2*, a homozygous viable hypomorph of Spi, had an abnormal cardiac phenotype (Table 2). Moreover, the homozygous mutant *Keren^exc27-7-B^* that lacks Keren, an EGFR ligand with similarity to Spitz that is involved in border cells in the *Drosophila* ovary had normal cardiac function [15], [39], [40] (Table 2).

These results suggest that the enlarged cardiac phenotypes observed in *Df(3L)ED4238*/+ and *ru^1^* homozygotes result from alterations in EGFR signaling since the abnormal cardiac phenotype can be rescued by cardiac-specific expression of soluble Spi. Furthermore, Spi may have a threshold effect on cardiac function since heterozygote loss of function alleles for *Spi* had a normal cardiac phenotype; but, the homozygous hypomorph, *spi-SCP2*, had an abnormal cardiac function [15], [38] (Table 2).

### Cardiac expression of dominant-negative EGFR causes post-developmental dilated cardiac chamber in adult *Drosophila*

Since restoration of EGFR signaling by transgenic expression of sSpi or EGFR rescued the abnormal cardiac phenotypes observed in *rho3* deficiencies and the *ru^1^* homozygotes, the effects of inhibiting EGFR signaling in the adult *Drosophila* heart were evaluated using a well-characterized dominant-negative EGFR (EGFR.DN) [16].

**Figure 6:**
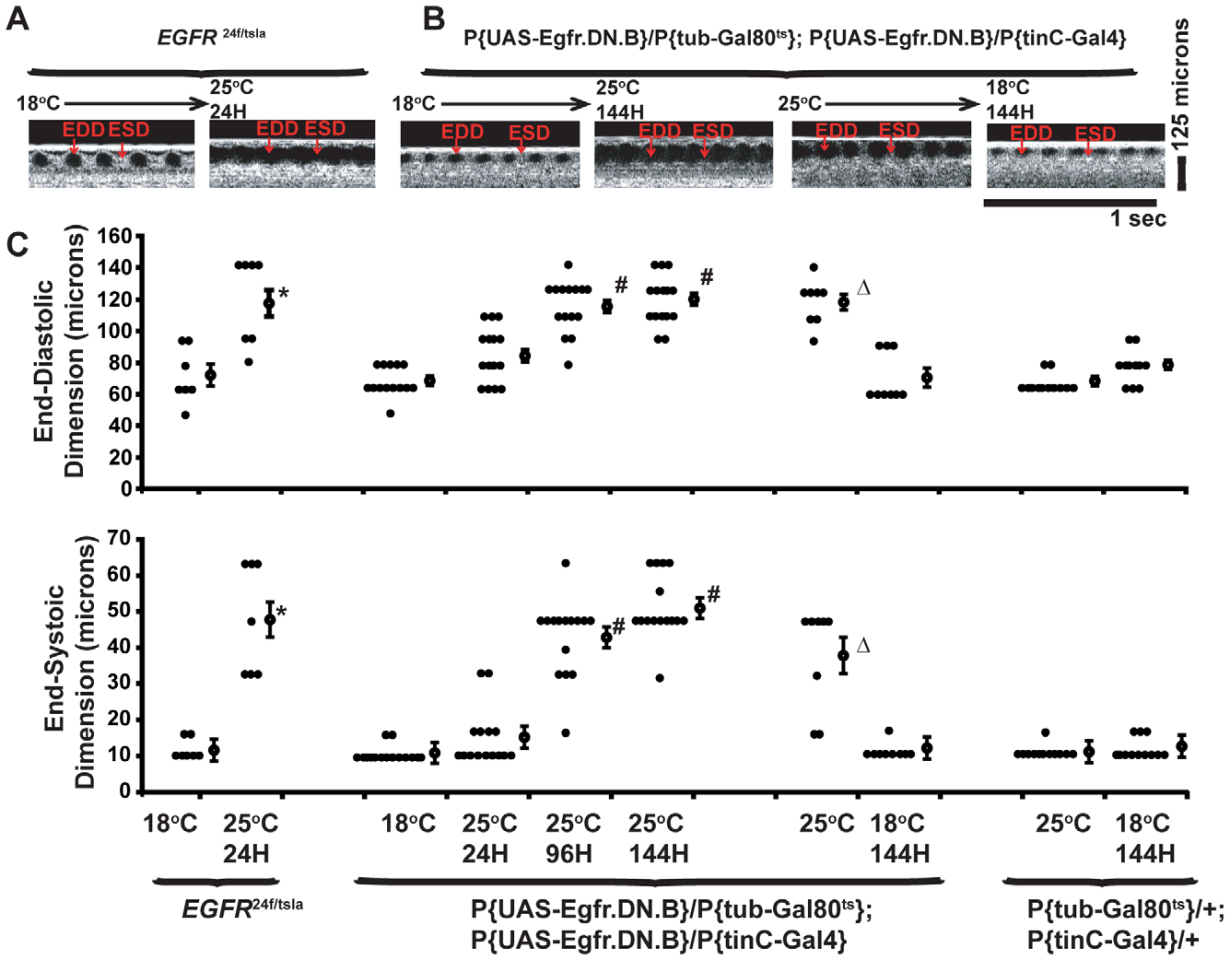
Cardiac expression of dominant negative EGFR (EGFR.DN) causes a progressive post-developmental enlarged heart and impaired systolic function in adult *Drosophila*. (A) Representative m-mode OCT images for *EGFR^f24^* ^/*tsla*^ at 18°C (baseline) and 24 hours after temperature shift to 25°C. Horizontal bar represents one second, 125 micron standard is shown. (B) Representative m-mode OCT images for p{UAS-EGFR.DN}/p{tub-Gal80^ts^}; p{UAS-EGFR.DN}/p{tinC-Gal4}, at 18°C (baseline) and 144 hours after temperature shift to 25°C or 25°C (baseline) and 144 hours after temperature shift to 18°C. OCT m-modes are from the same fly obtained at the indicated temperature and time intervals. Horizontal bar represents one second, 125 micron standard is shown. (C) Summary OCT data for end-diastolic dimension (EDD) (top) and end-systolic dimension (ESD) (bottom) for *EGFR^f24^* ^/*tsla*^ maintained at 18°C and after 24 hours at 25°C and p{UAS-EGFR.DN}/p{tubulin-Gal80^ts^}; p{UAS-EGFR.DN}/p{tinC-Gal4} maintained at 18°C followed by 24 hour, 96 hours, and 144 hours at 25°C. The summary also shows data for the reversal of the cardiac phenotype for p{UAS-EGFR.DN}/p{tubulin-Gal80^ts^}; p{UAS-EGFR.DN}/p{tinC-Gal4} maintained at 25°C then shifted to 18°C for 144 hours. Data for *EGFR^f24^*/*^tsla^* (n = 7) and p{UAS-EGFR.DN}/p{tubulin-Gal80^ts^}; p{UAS-EGFR.DN}/p{tinC-Gal4} (n = 15) experiments was obtained by serial OCT measurements in individual flies. The cardiac chamber sizes in p{tubulin-Gal80^ts^}/+; p{tinC-Gal4}/+ that did not harbor a UAS-EGFR.DN transgene were used as controls and examined at 18°C and 25°C. *p<0.05 for EGFR*^f24^* ^/*tsla*^ at 25°C versus 18°C baseline. #p<0.05 for p{UAS-EGFR.DN}/p{tubulin-Gal80^ts^}; p{UAS-EGFR.DN}/p{tinC-Gal4} at 25°C for 96 or 144 hours versus 18°C baseline; Δp<0.05 for p{UAS-EGFR.DN}/p{tubulin-Gal80^ts^}; p{UAS-EGFR.DN}/p{tinC-Gal4} at 18°C for 144 hours versus 25°C baseline. Closed circles represent individual measurements for each group, open circles represent the mean ± SEM for each group.

The cardiac function in flies with the hypomorphic allele, *EGFR^24f^*, in the context of a temperature sensitive lethal allele of EGFR (*EGFR^tsla^*) was evaluated by performing serial OCT cardiac measurements in individual flies at 18°C followed by 24 hours at 25°C [37]. Interestingly, the cardiac function in *EGFR^f24^* ^/*tsla*^ flies was similar to *w^1118^* at 18°C; however, the cardiac function deteriorated after 24 hr at 25°C (Figure 6A and 6C).

Next, the cardiac function was assessed by serial OCT measurements in individual flies that harbored UAS-EGFR.DN, tubulin-Gal80*^ts^*, and tinC-Gal4 [41]–[43]. Since the measurement of cardiac function by OCT is non-invasive and non-destructive, we serially measured the cardiac function in individual flies by performing OCT, allowing the fly to recover over a 24 hour period, and then repeating OCT assessments throughout the course of the experiments. Prior work in our laboratory demonstrated that the tubulin-Gal80*^ts^*; tinC-Gal4 fly driver line results in no significant transgene mRNA level at 18°C and between a 20 and 40 fold expression of transgene mRNA expression at 25°C by 24 to 48 hours [44]. Serial OCT cardiac evaluation in individual flies demonstrated that cardiac function was normal at 18°C but progressively deteriorated at 25°C as manifest by enlarged EDD and ESD (EDD 68 +/- 3 microns and ESD <10 microns (n = 15) at 18°C verses EDD 118 +/- 4 microns and ESD 51 +/- 2 microns (n = 15) at 25°C for 144 hr for P{UAS-EGFR.DN}/P{tubulin-Gal80^ts^}; P{UAS-EGFR.DN}/P{tinC-Gal4}) (Figure 6B and 6C). The effects of EGFR.DN expression on cardiac chamber size were similar in flies of 7 or 30 days age (data not shown). Interestingly, the EGFR.DN mediated effects on cardiac chamber size were reversible when the EGFR.DN expression was repressed by temperature shift (Figure 6B and 6C). The changes observed in cardiac chamber size and function depended on the expression of specific transgenes since flies that harbor tubulin-Gal80*^ts^*; tinC-Gal4 in the absence of UAS-EGFR.DN did not have abnormal cardiac phenotypes under similar temperature conditions (Figure 6C). Furthermore, flies that express unrelated UAS transgenes do not demonstrate alterations under similar temperature shift conditions [2], [44]. These results suggest that inhibition of EGFR signaling in the adult fly heart results in a progressive deterioration in cardiac function and suggests that proper EGFR signaling is required for the maintenance of normal adult heart function.

## Discussion

Our results suggest that rho3-mediated EGFR signaling is responsible for the enlarged cardiac phenotype observed in *Df(3L)ED4238*/+. Several lines of evidence support a role for rho3 in adult cardiac function. Analyses of the cardiac phenotypes in mutants that have *ru^1^* or *rho3^pLLB^* alleles are consistent with previously described effects in other tissues including the fly eye [13]. The *ru^1^* allele acts as a hypomorphic allele since homozygote *ru^1^*, but not heterozygote *ru^1^*, mutants have an enlarged cardiac chamber. However, homozygote and heterozygote *rho3^pLLB^* mutants have enlarged cardiac chambers consistent with the *rho3^pLLB^* allele acting as a null allele. The *ru^1^* allele encodes a premature stop codon however a truncated protein product was not detected and therefore the mechanism underlying the hypomorphic nature of the *ru^1^* allele is not clear. Possible explanations include a dosage effect such that a reduction in functional rho3 transcript/product to below a certain threshold results in a phenotype.

Prior work has demonstrated that rho3 interacts with rho1 and these proteases act together in cell signaling processes [14]. Although our results suggest that rho3 is important in the adult *Drosophila* heart, we recognize that rho1 and rho2 may also contribute to the complex signals that influence post-developmental cardiac function. Interestingly, *Df(3L)Exel6087*/+ that was heterozygote for a deficiency across the *rho1* and *rho2* loci but did not disrupt *rho3* had normal cardiac function. Additionally, *ru^1^/+*, *rho^7M43^*/+ flies that were heterozygous for the *ru^1^* allele and an amorphic allele of *rho1* also did not appear to have an enlarged cardiac chamber. QRT-PCR data also showed that rho1 mRNA levels were not significantly different in deficiency lines, *Df(3L)ED4238*/+ or *d07829-f07223*/+, that had an abnormal cardiac chamber size. Although the abnormal cardiac phenotype observed in strains that have heterozygote deficiencies in *rho3* or homozygous *ru^1^* mutants was rescued by tinC-mediated expression of recombinant *rho3*, we recognize that over-expression of rho3 may compensate for abnormalities in rho1 function and therefore obfuscate the importance of rho1 in adult cardiac function.

Our results also demonstrated that normal cardiac chamber size was restored in *Df(3L)ED4238*/+ and homozygous *ru^1^* by cardiac-specific expression of components downstream of rho3 including Spitz and EGFR. The restoration of cardiac function by transgenic expression of EGFR can be explained by two possible processes. First, the over-expression of EGFR monomers may increase the probability ligand-independent dimerization of EGFR resulting in potentially higher basal EGFR signaling and a rescue of cardiac function [10], [45]. Second, an unknown EGFR ligand other that Spitz may be responsible for restoration of cardiac function. Other EGFR ligands, including Keren and Gurkin, have been described to activate EGFR in specific tissues including the eye and ovary [39], [46]–[48]. Our results demonstrate that mutants with a null allele for Keren do not have an abnormal cardiac function although Keren was reported to function similarly to Spitz in eye development. However, other Spitz-like ligands may interact with cardiac EGFR and explain the observation that cardiac chamber size is normal in the presence of cardiac-specific EGFR transgene expression in the context of impaired rho3 function. Additionally, the rescue of the abnormal cardiac phenotype by tinC-Gal4 mediated transgenic expression of rho3 itself or active soluble Spitz suggests that these signals function in an autocrine manner or between the cardiomyocytes that comprise the cardiac tube.

Despite the functional abnormality observed by OCT in adult *ru^1^* homozygous mutants, we observed that the embryonic dorsal vessel and pupal heart morphology in *ru^1^* homozygotes did not differ from *w^1118^*. The adult homozygous *ru^1^* mutants had similar extra cardiac structures and dorsal diaphragm muscle fibers compared to *w^1118^*. Furthermore, the actin fiber organization did not appear obviously different from *w^1118^* although the heart chamber wall appeared thinner as compared to *w^1118^*. These findings are strikingly different from other fly mutants that have enlarged cardiac chambers [5], [31], [49]. For example, *hdp^2^*, a fly that has a recessive point mutation in troponin-I, has a markedly enlarged heart with poor systolic function and has abnormalities in the organization of contractile elements. Additionally, flies that have deficiencies in dystrophin also demonstrate poor contractile function, enlarged cardiac chambers, and myofibrillar disorganization [31]. Our findings suggest that additional mechanisms may exist that responsible for an enlargement in the *Drosophila* heart and are not associated with dramatic abnormalities in myofibrillar organization or cardiomyocyte cell number. While the exact mechanisms through which alterations in rhomboid signaling translate into functional myocardial impairment remain to be elucidated, our results support that further studies of the downstream signaling components of EGFR are necessary.

The post-developmental expression of a dominant-negative EGFR under the control of the temperature-sensitive Gal80 system resulted in a progressive deterioration in cardiac function. These results suggest that rho3-EGFR signaling maintains adult cardiac function. Additionally, the developing cardiac system in *Drosophila* appears to be sensitive to the level of EGFR activation since tinC-mediated expression of constitutively-active EGFR transgenes resulted in the failure of flies to eclose from pupal cases. We also observed the rescue of cardiac chamber size and function post-developmentally when EGFR-DN was repressed in adult flies after the development of an enlarged heart. The findings suggest that the adult fly heart has a degree of plasticity such that the removal of a deleterious cardiac perturbation may restore normal heart camber size and function. This is quite different from most cardiomyopathies in mammals in which a defect in cardiac function often leads to a progressive and irreversible deterioration of the heart. The mechanisms underlying the plasticity of the adult fly heart and the exact downstream components of EGFR signaling that are responsible for maintaining adult cardiac function will require further investigation.

The identification of EGFR signaling in adult *Drosophila* heart function underscores the concept that evolutionarily conserved signaling mechanisms are required to maintain normal myocardial function. In mammals the EGF receptor is one member of the larger ErbB receptor family. Recently, Alvarado et al. had shown that human ErbB2 is the closest structural relative to *Drosophila* EGFR and suggest that ErbB2 shares more similarities with invertebrate EGFR than other isoforms of other human ErbB members [50]. ErbB2 is a target of chemotherapy in the treatment of several cancers and the inhibition of ErbB2 signaling by transgenic knock-out in mice and herceptin-treatment humans is associated with the development of dilated cardiomyopathy [51]–[53]. Our findings support that EGFR signaling is required for cardiac function in *Drosophila*. Furthermore, our results in conjunction with the demonstration that altered ErbB2 signaling underlies certain forms of mammalian cardiomyopathy suggest that an evolutionarily conserved signaling mechanism may be necessary to maintain post-developmental cardiac function.

Rhomboid proteases are highly conserved and present throughout the animal kingdom including mammals [54]. Although rhomboids do not appear be involved in EGF signaling in mammals, prior work has identified ephrin B and thrombomodulin as potential substrates of rhomboids [55], [56]. Our results demonstrate a new role for rhomboid proteases in post-developmental adult *Drosophila* cardiac function and suggest that further investigations may provide insight into potential mechanisms of mammalian rhomboid proteases in human dilated cardiomyopathies and heart failure.

Our observations also support an important concept that underlies our screening strategy. Namely, a screen for altered cardiac chamber dimensions and function in adult flies based on functional phenotyping can identify mutations that do not significantly alter the morphology of the dorsal vessel in the embryo. We acknowledge that the consequences of cardiac dysfunction in the fly are not the same as in humans; however, aspects of cardiac abnormalities are shared at some level and may help identify pathways that potentially affect human disease. While this concept may be simple, our observations support the rationale of our ongoing screens to identify genes that cause cardiac abnormalities in adult *Drosophila*.

## Materials and Methods

### Materials

DrosDel and Exelixis stocks as well as all mutants were obtained from the Bloomington *Drosophila* Stock Center unless otherwise stated below. All stocks were maintained on standard yeast protein media at room temperature. The p{tinC-Gal4} was kindly proved by Manfred Frasch [22]. The p{UAS-sSpi} and p{UAS-mSpi} stocks were kindly provided by Andreas Bergmann and Hermann Steller [10], [36]. The p{tubulin-Gal80^ts^}; p{tinC-Gal4} stocks were engineered based on methods described by McGuire *et. al.* [42]. *rho-3^PLLb^*, *spi-SCP2*, *rho-4*, *rho-6*, and the double *rho-4;rho-6* mutants were kindly provided by Matthew Freeman [13]. The *Keren^exc 27-7-B^* mutant was kindly provided by Denise Montell [39].

### Generation of specific genomic deficiencies

*Drosophila* containing specific P-element insertions designated P{XP}Ptp61F^d01050^, PBac{WH}Ptp61F^f07223^, P{XP}Ptp61F^d07829^, and PBac{WH}msd5^f05423^ were obtained from the Exelixis Collection at Harvard Medical School. Specific genomic deficiencies were generated using P{hsFLP}1, y[1] w[1118]; Dr[Mio]/TM3, ry[*] Sb[1] according to the established methods [6]. The genomic deficiencies were genotyped using iPCR methods and DNA sequencing as described by Parks *et. al.* [6].

### OCT measurement of cardiac function in adult *Drosophila*

Cardiac function in adult *Drosophila* was measured using a custom built OCT microscopy system (Bioptigen, Inc. Durham, NC) as previously described [5]. Flies that had genomic deficiencies from the Exelixis or DrosDel collections were bred with *w^1118^* to remove balancer chromosomes and the F1 generation was used to examine cardiac parameters. Additionally, all stocks that had alleles maintained over a balancer chromosomes were bred to *w^1118^* to remove the balancer chromosome prior to evaluation of the cardiac parameters in the F1 offspring.

Briefly, adult female *Drosophila* between 7 and 10 days post eclosion were briefly subjected to CO_2_, placed on a soft gel support, and allowed to fully awaken based on body movement. All flies were imaged as B-modes in the longitudinal orientation to identify the cardiac chamber in the A1 segment and then in the transverse orientation to center the heart chamber. Multiple 3 second OCT m-modes were recorded and images were processed using ImageJ software using a 125 micron standard. After m-mode acquisition, the flies were examined in the transverse B-mode orientation to assure consistent measurements from the heart chamber. End-diastolic (EDD), end-systolic (ESD), and heart rate were determined from 3 consecutive heart beats. Fractional shortening (FS) was calculated as [EDD-ESD]/EDD ×100. OCT measurements were binned into 8 micron cut-off values based on the axial resolution of the OCT instrument. Each OCT measurement is represented in the graphs as a closed circle to provide an estimate of the frequency observed for each measurement in the corresponding groups for the experiments described in the text. A summary of the OCT data for each group represented by an open circle and corresponds to the mean +/- SE. Since the resolution of OCT is limited to 8 microns, we also analyzed the data in a dichotomized manner where we defined an “enlarged” heart as an EDD >90 microns and “impaired systolic function” as an ESD >20 microns.

For serial OCT studies, adult flies were bred at 18°C until 7 to 10 days post eclosion prior to initial evaluation of cardiac function by OCT. After each OCT measurement, individual files were gently removed from the soft gel and placed in individual vials at 25°C with food. Serial evaluation of cardiac function by OCT was conducted as described in the figures.

### QRT-PCR

Total RNA samples from ten female flies of 7 days post-eclosion were prepared from *w^1118^*, *Df(3L)ED4328*/+, heterozygous genomic deficiencies *d07829-f07223*/+ or *f05432-d01050*/+, *ru^1^* homozygous mutants (*ru^1^/ru^1^*), *rho3^pLLB^* heterozygotes (*rho3^pLLB^/+*), or *rho3^pLLB^* homozygotes (*rho3^pLLB^/rho3^pLLB^*) using RNA-Bee (Tel-Test “B”). Embryos, larvae, pupae, and dissected adult tissues were collected from *w^1118^* to define the transcriptional expression pattern of rho1 and rho3. Two µg of RNA was used for generation of cDNA using SuperScript II reverse transcriptase (Invitrogen, Inc.). Applied Biosystems Taqman Gene expression assays were used to perform quantitative (real time) RT-PCR (rho3: Dm01837284_m1; rho1: Dm01821932_m1, and Ribosomal Protein L32 (Rpl32): Dm 02151827-g1 for endogenous control). The following reaction components were used for each probe: 2 µL cDNA, 12.5 µl 2X TaqMan Universal PCRMaster Mix without Amperase (Applied Biosystems, Inc.), 1.25 µl of probe, and 9.25 µl water in a 25 µl total volume. Reactions were amplified and analyzed in triplicate using an ABI PRISM*H* 7000 Sequence Detection System. PCR reaction conditions were as follows: Step 1: 95°C for 10 minutes, Step 2: 40 cycles of 95°C for 15 seconds followed by 60°C for 1 minute. Expression relative to Rpl32 was calculated using 2^−ΔΔCt^ and levels were normalized to baseline. We performed three independent experiments in triplicate using different batches of flies each time.

### Transgenic wt-*rho3* and tinC-GFP lines

The cDNA encoding wt-*rho3* was isolated by RT-PCR from *w^1118^* adult flies, subcloned into pUAST, and verified by sequencing. Transgenic *Drosophila* harboring wt-*rho3* were generated by established methods [57]. The tinC-GFP *Drosophila* lines were created by isolating and subcloning the 304 bp tinC genomic sequence from *w^1118^* into pGreen-H-Pelican [22], [58].

### Expression of rho3 cDNA in S2 cells

S2 cells were maintained in Schneider's media containing 10% fetal bovine serum under standard conditions. Total RNA was isolated from adult *w^1118^*, *Oregon-R*, and homozygous *ru^1^* mutant flies using RNAzole-Bee (Tel-Test “B”) and then used to obtain cDNA by a reverse transcriptase mediated reaction using oligo-d(T)_16_ primers. Next, *rho3* cDNA was isolated by PCR with primers corresponding to the 5′ and 3′ ends of *rho3* and Platinum-Taq (Invitrogen Inc.). The PCR products were subcloned into pUAST with 5′ primers containing flag-tag epitopes. DNA sequencing was performed at multiple steps throughout the subcloning steps.

S2 cells were transfected with 1 µg of pUAST alone or pUAST containing N-terminally flag-tagged rho3 cloned from *w^1118^*, *Oregon-R*, and *ru^1^* mutant flies, respectively, in the presence of 1 ug of pPTGAL plasmid encoding Gal4 driven by an ubiquitin promoter (gift from H. Amerin) using Cellfectin reagent (Invitrogen, Inc.). After 48 hours of incubation, the cells were washed gently in PBS, lysed in 500 µl of RIPA buffer, placed on ice for 20 minutes, and then subjected to centrifugation at 12,000×g for 15 minutes. Total protein content in each lysate was determined using a Bio-Rad protein assay. 20 µg of total protein was applied to 12% polyacrylamide denaturing gels and proteins were resolved by electrophoresis prior to Immunoblotting. The mouse monoclonal anti-flag M2 antibody (1∶2000) (Sigma, Inc.) was used to detect Flag-fusion proteins.

### Evaluation of adult cardiac morphology

Adult *Drosophila* corresponding to homozygous tinC-GFP in *w^1118^* background alone or in the presence of homozygous *ru^1^* at 7 days age post eclosion were used to examine adult cardiac morphology. Flies were briefly anesthetized by administration of CO2, the head and thorax were removed and the abdomen was placed in artificial hemolymph buffer (108 mM Na^+^, 5 mM K^+^, 2 mM Ca^2+^, 8 mM MgCl_2_, 1 mM NaH_2_PO_4_, 4 mM NaHCO_3_, 10 mM sucrose, 5 mM trehalose, and 5 mM Hepes (pH 7.1) [59]. An incision was made along the ventral aspect of the abdomen and the internal abdominal organs were gently removed. The surrounding fat and tissue was removed using a pulled glass capillary pipette. Then hemolymph buffer that contained 10mM EGTA was added to relax the cardiac muscle as described by Alayari *et. al.* [60]. Next, samples were fixed in 4% paraformaldehyde for 20 minutes at room temperature prior to staining with anti-GFP-antibody (1∶500) (Invitrogen, Inc.) for detection of cardiomyocytes and phalloidin-TexasRed (1∶1,000) (Invitrogen, Inc.) for actin staining. The stained heart preparations were visualized under a Zeiss LSM510 confocal microscope and 0.4 micron Z-stack images were analyzed.

For evaluation of cardiac morphology during pupal stages, *Drosophila* corresponding to homozygous tinC-GFP in *w^1118^* background or in the presence of homozygous *ru^1^* were collected between the ∼P6 and P13 stages according to staging described by Bainbridge and Bownes [61]. Pupal hearts were directly visualized using a Leica M165FC fluorescent stereomicroscope.

For heart rate measurements, whole flies corresponding to homozygous tinC-GFP in *w^1118^* background or in the presence of homozygous *ru^1^*were examined intact or after dissection as described above and the hearts were visualized using a Leica M165FC fluorescent stereomicroscope equipped with a DFC310Fx camera. Images were captures at a frame rate of ∼38 fps for analysis.

### Histological analysis

Adult female flies of 7 to 10 days age were collected, immersed in 70% alcohol for 1 minute, and then fixed in 10% buffered formalin overnight at 4°C. Next, the specimens were rinsed in PBS and dehydrated in ethanol through sequential gradients. Then, the samples were washed twice with xylenes before immersion in liquid paraffin. After solidification, paraffin blocks were sectioned serially at 8 µm thickness in longitudinal or transverse orientation (n = 8 flies per orientation per group). Sections were rehydrated and stained with hematoxylin and eosin. The following criteria were used to control for the position of the heart chamber among different flies that were evaluated. First, we identified the transverse section with that contained the *en face* view of the ventral longitudinal fibers (VLF) corresponding to the anterior conical chamber. Second, we counted three serial 8 micron sections posterior from the section with the *en face* view of the VLF. Third, we measured the chamber dimension in the next three serial 8 micron sections (denoted MS1, MS2, and MS3 in Figure S4). Sections were analyzed using an Olympus 1X70 microscope at 400× magnification and images captured using a PaxCam digital camera. We measured the diameter of the cardiac chamber in the dorsal to ventral direction in transverse oriented sections since this measurement corresponded to the orientation of the measurements obtained by OCT. Wall thickness was calculated by measuring the cardiac chamber wall width along the lateral walls at two positions in three serial sections to obtain the mean ± SEM.

To determine the degree that fixation may have had on chamber size, we examined the chamber size in flies that were fixed after the 70% alcohol step by performing measurements using OCT. The cardiac chamber sizes in fixed flies were similar to the EDD in awake, adult flies.

### Statistical analysis

Comparisons of EDD chamber dimensions were determined by either a student's t-test for two samples or an analysis of variances (ANOVA) with Bonferroni corrections for multiple comparisons when necessary. Comparisons of ESD chamber dimensions were determined by Mann-Whitney tests for comparisons of two samples or Kruskal-Wallis tests with a Dunn's test for multiple comparisons. GraphPad Prism statistical software (GraphPad Software Inc.) was used for all analyses.

## Supporting Information

Figure S1 Fractional Shortening measurements in adult *Drosophila* as determined by OCT. (A) Summary data of cardiac measurements for Fractional Shortening (FS) from *w^1118^*, *Df(3L)ED4238*/+, and *hdp^2^*. n = 13−17 flies per group. *p<0.05 for the indicated measurements compared to *w^1118^* using an ANOVA with Bonferroni correction. (B) Summary data of cardiac measurements for FS from *w^1118^*, *ru^1^* homozygotes (designated *ru^1^/ru^1^*), *ru^1^* heterozygotes (*ru^1^*/+), *rho3^pLLB^* heterozygotes (*rho3^pLLB^/+*), and *rho3^pLLB^* homozygotes (*rho3^pLLB^/rho3^pLLB^*). n = 8−17 flies per group. *p<0.05 for the indicated measurements by ANOVA with Bonferroni correction for multiple comparisons. (C) Summary data of cardiac measurements for FS from *w^1118^*, homozygous *ru^1^* mutants (*ru^1^*/*ru^1^*), *Df(3L)ED4238*/+, and transgenic flies harboring the driver *tinC*-Gal4 and UAS-wt-*rho3*, UAS-*mSpi,* UAS-*sSpi, or* UAS-*EGFR* in the context of the *Df(3L)ED4238*/+ or the homozygous *ru^1^* mutant. n = 8−17 flies per group. *p<0.05 for the indicated measurements comparing *Df(3L)ED4238*/+ alone versus Df(3L)ED4238/+ expressing the indicated transgenes or homozygous *ru^1^* alone versus homozygous *ru^1^* in the context of the indicated transgenes. FS measurements were analyzed by student’s t-test. (D) Summary OCT data for FS for *EGFR^f24^* ^/*tsla*^ maintained at 18°C and after 24 hours at 25°C and p{UAS-EGFR.DN}/p{tubulin-Gal80^ts^}; p{UAS-EGFR.DN}/p{tinC-Gal4} maintained at 18°C followed by 24 hour, 96 hours, and 144 hours at 25°C. The summary also shows data for the reversal of the cardiac phenotype for p{UAS-EGFR.DN}/p{tubulin-Gal80^ts^}; p{UAS-EGFR.DN}/p{tinC-Gal4} maintained at 25°C then shifted to 18°C for 144 hours. Data for *EGFR^f24^*/*^tsla^* (n = 7) and p{UAS-EGFR.DN}/p{tubulin-Gal80^ts^}; p{UAS-EGFR.DN}/p{tinC-Gal4} (n = 15) experiments was obtained by serial OCT measurements in individual flies. The fractional shortening in p{tubulin-Gal80^ts^}/+; p{tinC-Gal4}/+ that did not harbor a UAS-EGFR.DN transgene were used as controls and examined at 18°C and 25°C. *p<0.05 for EGFR*^f24^* ^/*tsla*^ at 25°C versus 18°C baseline. #p<0.05 for p{UAS-EGFR.DN}/p{tubulin-Gal80^ts^}; p{UAS-EGFR.DN}/p{tinC-Gal4} at 25°C for 96 or 144 hours versus 18°C baseline; Δp<0.05 for p{UAS-EGFR.DN}/p{tubulin-Gal80^ts^}; p{UAS-EGFR.DN}/p{tinC-Gal4} at 18°C for 144 hours versus 25°C baseline. Closed circles represent individual measurements for each group and open circles represent the mean ± SEM for each group.
(0.65 MB TIF)

Figure S2 The mutant *ru^1^* encodes a missense mutation. (A) Representative DNA sequencing chromatograms for *rho3* from *w^1118^* and *ru^1^* showing that *ru^1^* has a premature stop codon at nucleotide position 163 corresponding to amino acid position 55. A schematic of the gene structure is shown below with coding exons #4 and 5 (gray boxes) and introns (black line). The asterisk represents the position of the mutation in *ru^1^*. (B) Immunoblot of 20 ug total protein lysate from S2 cells expressing N-terminal Flag-tagged rho3 corresponding to cDNAs from *w^1118^*, *Oregon-R*, and *ru^1^* flies. Each construct was expressed from 1 ug of corresponding pUAST constructs co-transfected in S2 cells with 1 ug of a pPTGAL plasmid encoding Gal4 driven by an ubiquitin promoter. rho3 protein produce is denoted by the arrow and the asterisk denotes a non-specific protein band. Protein markers in kDa are shown on left.
(0.58 MB TIF)

Figure S3 Cardiac morphology in *w^1118^* and homozygous *ru^1^* mutants during the pupal stage. Panels are representative of hearts from transgenic *Drosophila* that express tinC-GFP in the context of *w^1118^* (A–C) or homozygous *ru^1^* mutants (D–E). (A,D) represent ∼P6, (B,E) represent ∼P8, and (C,F) represent ∼P13 pupae based on staging described by Bainbridge and Bownes [61]. The inset below each panel shows a magnified view of the tinC-GFP positive heart. The A1, A2, A3, and A4 segments are denoted in each panel and the A5 segment could not be visualized. N = 45 for *w^1118^* pupae and N = 47 for homozygous *ru^1^* mutant pupae for each group.
(7.64 MB TIF)

Figure S4 Morphology of the adult heart in homozygous *ru^1^* mutants. (A) Schematic representation of an adult *Drosophila* heart in the longitudinal orientation showing the region corresponding to serial 8 microns sections for histological analyses. The transverse section showing the cardiac chamber (CC) and the *en face* view of the ventral longitudinal fibers (VLF) was used to orient the heart position among the flies analyzed. The cardiac chamber size from the mid dorsal to ventral wall was measured in three serial sections (MS1, MS2, and MS3) to obtain the mean ± SEM. The red box indicated the approximate region of the cardiac chamber that was evaluated in the corresponding transverse sections. (B) Representative H&E stained sections in the longitudinal (top) and transverse orientation (bottom) through the adult cardiac chamber in *w^1118^* and homozygous *ru^1^* mutants (*ru^1^/ru^1^*). (n =  8 per group). A 125 micron standard is shown. (C) Summary data for cardiac chamber size and cardiac wall thickness for *w^1118^* and homozygous *ru^1^* mutants (*ru^1^/ru^1^*) (n = 8 flies analyzed for each group). The cardiac chamber size was measured along the vertical axis from the mid dorsal to mid ventral walls and the wall thickness was measured from the mid lateral walls in sections corresponding to MS1, MS2, and MS3 in (A). The values are expressed as the mean ± SEM in microns. *p<0.05 for chamber size or wall thickness analyzed by students t-tests.
(3.32 MB TIF)

Figure S5 Heart rates in *w^1118^* and homozygous *ru^1^* mutants are similar. The heart rates were measured at one day or seven to fourteen days after eclosion in intact flies that expressed GFP driven by tinC in the context of control (*w^1118^*) or homozygous *ru^1^* alleles. Heart rates were also measured in dissected specimens perfused in artificial hemolymph at seven days after eclosion. n =  4−11 individual flies.
(0.40 MB TIF)

Table S1 Cardiac parameters from the initial screen of genomic deficiencies along *Drosophila* Chromosome 3L. The data represent the cardiac measurements in adult flies obtained by OCT for end-diastolic dimension (EDD) in microns, end-systolic dimension (ESD) in microns, and fractional shortening (FS). FS was calculated as (EDD-ESD)/EDD x 100. Values are expressed as the mean +/- SE. N = 14−16 samples per group. Additionally, the data is represented in a binary manner with “Dilated” defined as an EDD > 90 microns and “Impaired Systolic Function” defined as an ESD > 20 microns) where “N”  = no and “Y” = yes.
(0.07 MB DOC)

Table S2 Expression of *rho1* and *rho3* mRNA in different developmental stages and adult tissues. Representative quantitative real-time RT-PCR. Total RNA and cDNA were prepared from embryos in indicated time points after birth, larvae, pupae, and dissected adult tissues of *w^1118^*. Summary data show relative gene expression of *rho3* and *rho1* at developmental stages and in adult tissues (expressed as fold-change compared to the *rho3 or rho1* expression level at 0–8h embryo, respectively). Data represent mean ± SE of at least three independent experiments, each performed in triplicate.
(0.03 MB DOC)
