# Supplementary figures and images for: Affecting Rhomboid-3 Function Causes a Dilated Heart in Adult Drosophila
Source: PLoS Genet. 2010 May 27;6(5):e1000969. doi: 10.1371/journal.pgen.1000969 (PMC2877733; doi:10.1371/journal.pgen.1000969)

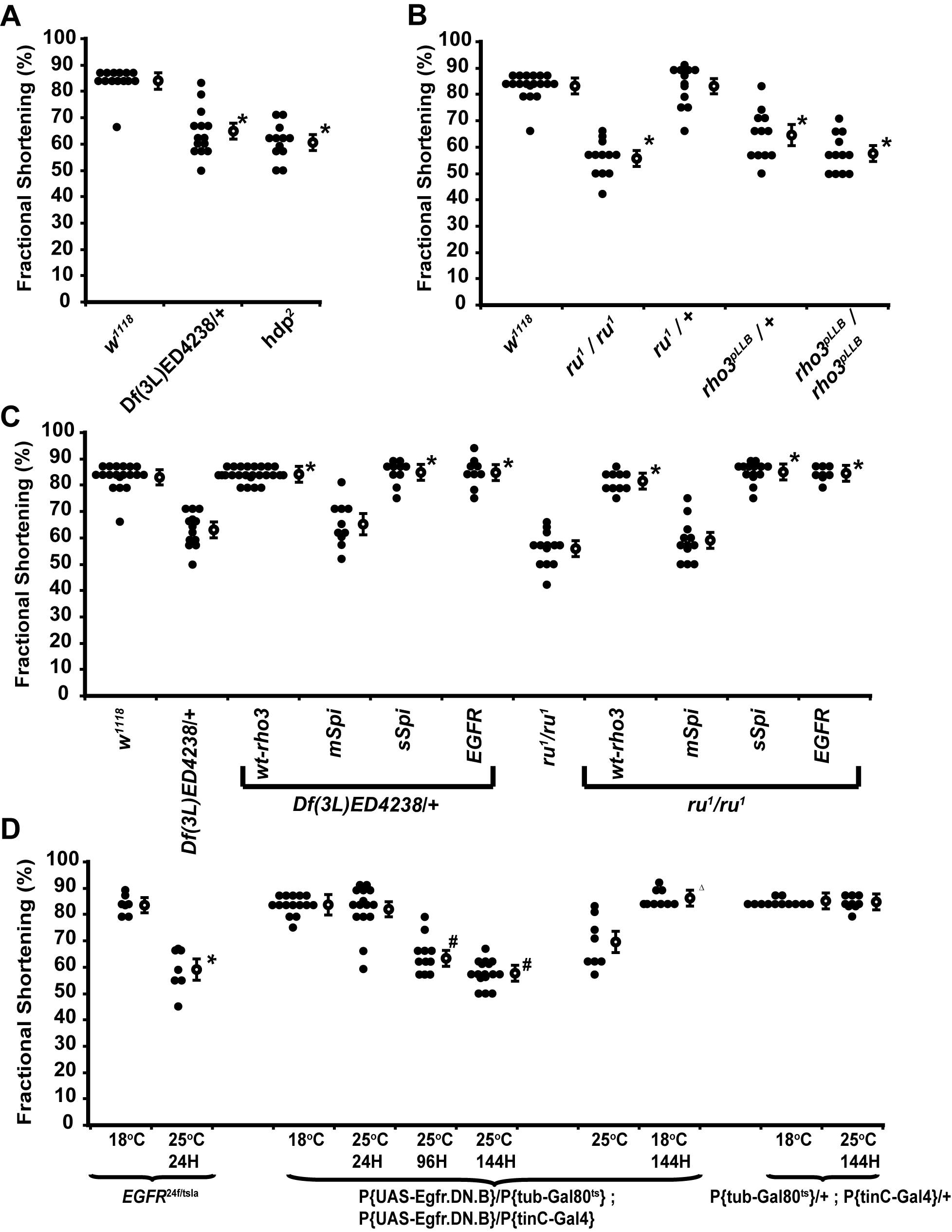

Supplement: Figure S1 — Fractional Shortening measurements in adult Drosophila as determined by OCT. (A) Summary data of cardiac measurements for Fractional Shortening (FS) from w1118, Df(3L)ED4238/+, and hdp2. n = 13−17 flies per group. *p<0.05 for the indicated measurements compared to w1118 using an ANOVA with Bonferroni correction. (B) Summary data of cardiac measurements for FS from w1118, ru1 homozygotes (designated ru1/ru1), ru1 heterozygotes (ru1/+), rho3pLLB heterozygotes (rho3pLLB/+), and rho3pLLB homozygotes (rho3pLLB/rho3pLLB). n = 8−17 flies per group. *p<0.05 for the indicated measurements by ANOVA with Bonferroni correction for multiple comparisons. (C) Summary data of cardiac measurements for FS from w1118, homozygous ru1 mutants (ru1/ru1), Df(3L)ED4238/+, and transgenic flies harboring the driver tinC-Gal4 and UAS-wt-rho3, UAS-mSpi, UAS-sSpi, or UAS-EGFR in the context of the Df(3L)ED4238/+ or the homozygous ru1 mutant. n = 8−17 flies per group. *p<0.05 for the indicated measurements comparing Df(3L)ED4238/+ alone versus Df(3L)ED4238/+ expressing the indicated transgenes or homozygous ru1 alone versus homozygous ru1 in the context of the indicated transgenes. FS measurements were analyzed by student’s t-test. (D) Summary OCT data for FS for EGFRf24 /tsla maintained at 18°C and after 24 hours at 25°C and p{UAS-EGFR.DN}/p{tubulin-Gal80ts}; p{UAS-EGFR.DN}/p{tinC-Gal4} maintained at 18°C followed by 24 hour, 96 hours, and 144 hours at 25°C. The summary also shows data for the reversal of the cardiac phenotype for p{UAS-EGFR.DN}/p{tubulin-Gal80ts}; p{UAS-EGFR.DN}/p{tinC-Gal4} maintained at 25°C then shifted to 18°C for 144 hours. Data for EGFRf24/tsla (n = 7) and p{UAS-EGFR.DN}/p{tubulin-Gal80ts}; p{UAS-EGFR.DN}/p{tinC-Gal4} (n = 15) experiments was obtained by serial OCT measurements in individual flies. The fractional shortening in p{tubulin-Gal80ts}/+; p{tinC-Gal4}/+ that did not harbor a UAS-EGFR.DN transgene were used as controls and examined at 18°C and 25°C. *p<0.05 for [file pgen.1000969.s001.tif]

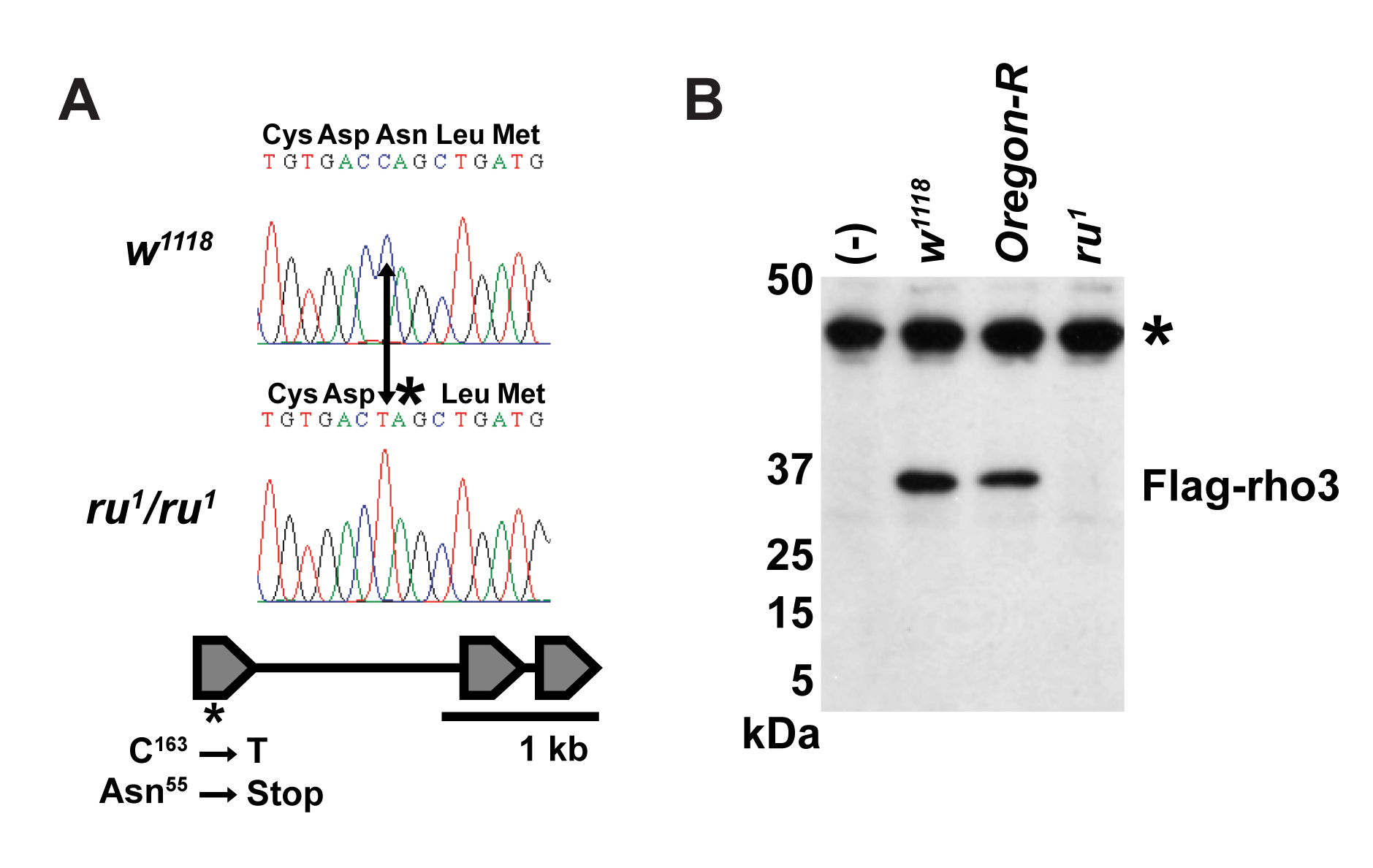

Supplement: Figure S2 — The mutant ru1 encodes a missense mutation. (A) Representative DNA sequencing chromatograms for rho3 from w1118 and ru1 showing that ru1 has a premature stop codon at nucleotide position 163 corresponding to amino acid position 55. A schematic of the gene structure is shown below with coding exons #4 and 5 (gray boxes) and introns (black line). The asterisk represents the position of the mutation in ru1. (B) Immunoblot of 20 ug total protein lysate from S2 cells expressing N-terminal Flag-tagged rho3 corresponding to cDNAs from w1118, Oregon-R, and ru1 flies. Each construct was expressed from 1 ug of corresponding pUAST constructs co-transfected in S2 cells with 1 ug of a pPTGAL plasmid encoding Gal4 driven by an ubiquitin promoter. rho3 protein produce is denoted by the arrow and the asterisk denotes a non-specific protein band. Protein markers in kDa are shown on left. (0.58 MB TIF) [file pgen.1000969.s002.tif]

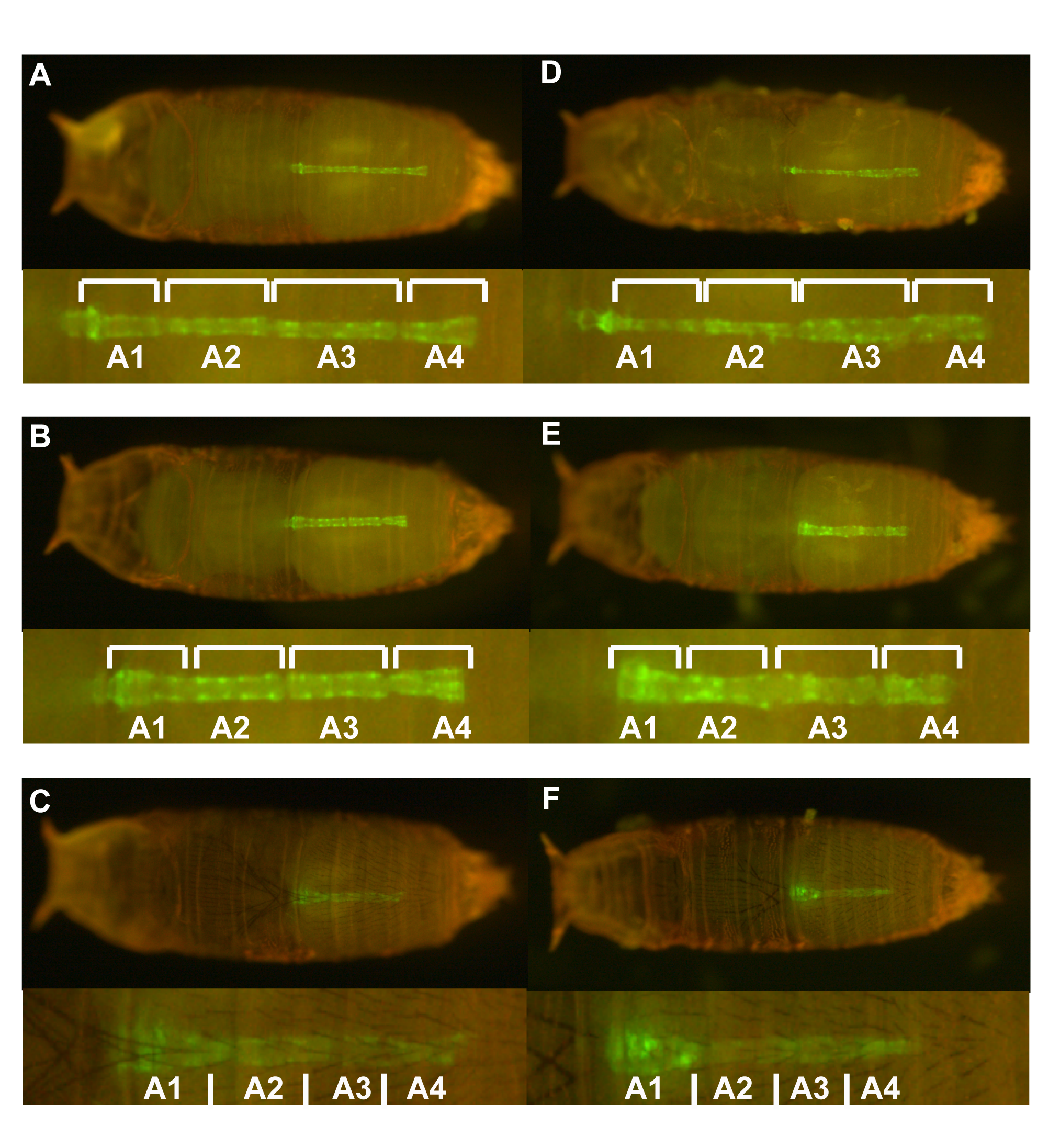

Supplement: Figure S3 — Cardiac morphology in w1118 and homozygous ru1 mutants during the pupal stage. Panels are representative of hearts from transgenic Drosophila that express tinC-GFP in the context of w1118 (A–C) or homozygous ru1 mutants (D–E). (A,D) represent ∼P6, (B,E) represent ∼P8, and (C,F) represent ∼P13 pupae based on staging described by Bainbridge and Bownes [61]. The inset below each panel shows a magnified view of the tinC-GFP positive heart. The A1, A2, A3, and A4 segments are denoted in each panel and the A5 segment could not be visualized. N = 45 for w1118 pupae and N = 47 for homozygous ru1 mutant pupae for each group. (7.64 MB TIF) [file pgen.1000969.s003.tif]

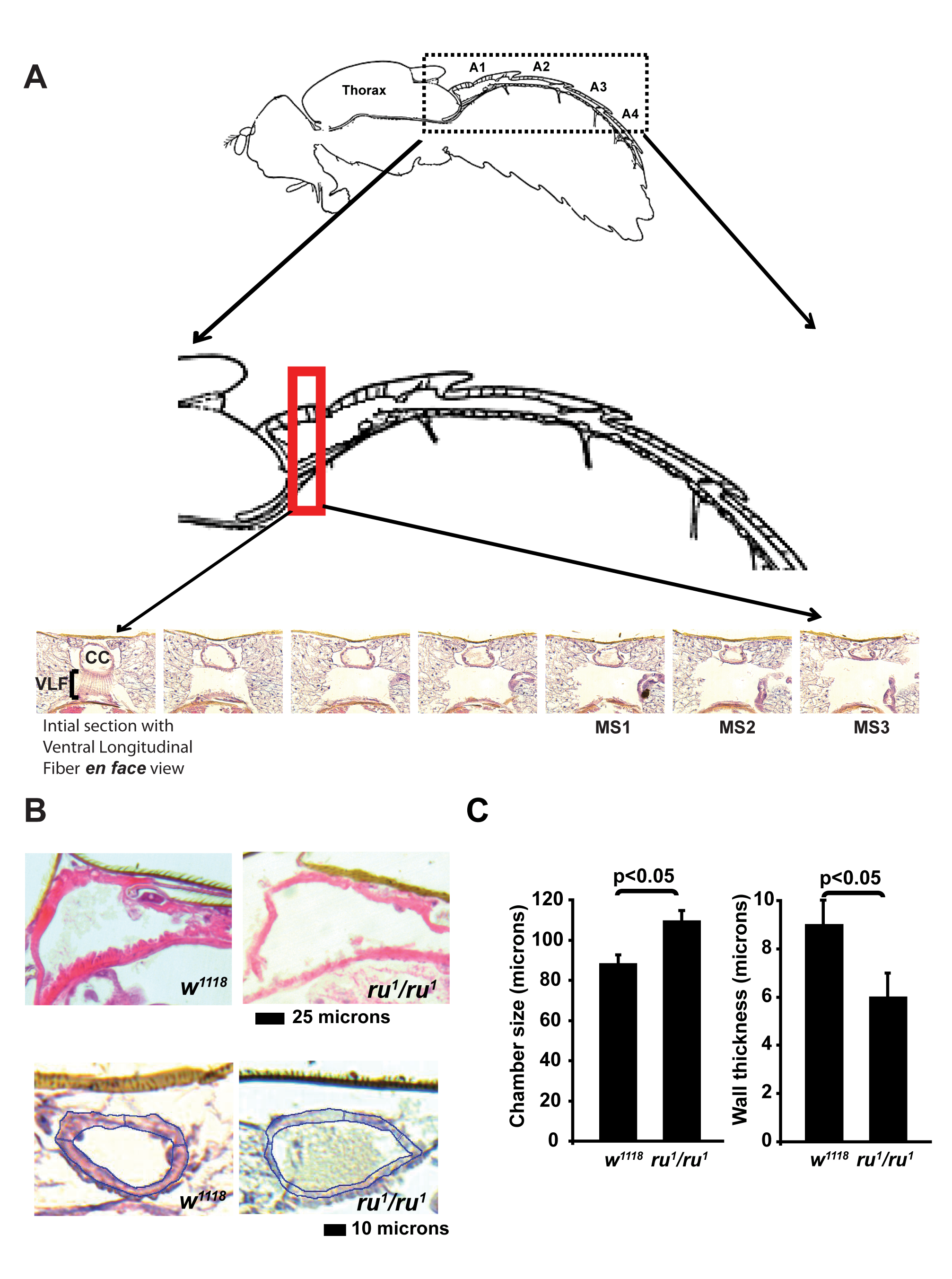

Supplement: Figure S4 — Morphology of the adult heart in homozygous ru1 mutants. (A) Schematic representation of an adult Drosophila heart in the longitudinal orientation showing the region corresponding to serial 8 microns sections for histological analyses. The transverse section showing the cardiac chamber (CC) and the en face view of the ventral longitudinal fibers (VLF) was used to orient the heart position among the flies analyzed. The cardiac chamber size from the mid dorsal to ventral wall was measured in three serial sections (MS1, MS2, and MS3) to obtain the mean ± SEM. The red box indicated the approximate region of the cardiac chamber that was evaluated in the corresponding transverse sections. (B) Representative H&E stained sections in the longitudinal (top) and transverse orientation (bottom) through the adult cardiac chamber in w1118 and homozygous ru1 mutants (ru1/ru1). (n = 8 per group). A 125 micron standard is shown. (C) Summary data for cardiac chamber size and cardiac wall thickness for w1118 and homozygous ru1 mutants (ru1/ru1) (n = 8 flies analyzed for each group). The cardiac chamber size was measured along the vertical axis from the mid dorsal to mid ventral walls and the wall thickness was measured from the mid lateral walls in sections corresponding to MS1, MS2, and MS3 in (A). The values are expressed as the mean ± SEM in microns. *p<0.05 for chamber size or wall thickness analyzed by students t-tests. (3.32 MB TIF) [file pgen.1000969.s004.tif]

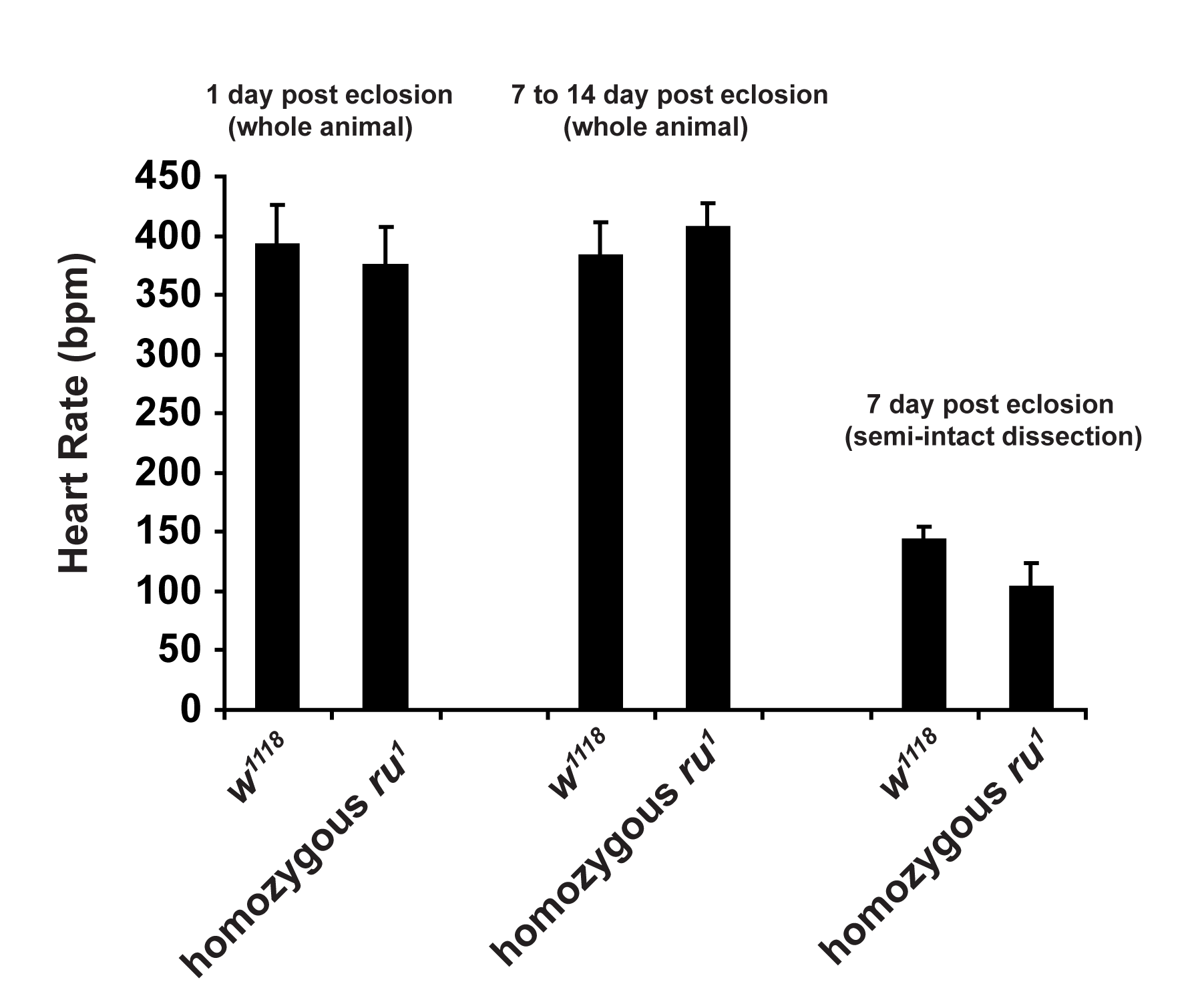

Supplement: Figure S5 — Heart rates in w1118 and homozygous ru1 mutants are similar. The heart rates were measured at one day or seven to fourteen days after eclosion in intact flies that expressed GFP driven by tinC in the context of control (w1118) or homozygous ru1 alleles. Heart rates were also measured in dissected specimens perfused in artificial hemolymph at seven days after eclosion. n = 4−11 individual flies. (0.40 MB TIF) [file pgen.1000969.s005.tif]
