# Supplementary material for: Affecting Rhomboid-3 Function Causes a Dilated Heart in Adult Drosophila
Source: PLoS Genet. 2010 May 27;6(5):e1000969. doi: 10.1371/journal.pgen.1000969 (PMC2877733; doi:10.1371/journal.pgen.1000969)
Supplement: Table S1 — Cardiac parameters from the initial screen of genomic deficiencies along Drosophila Chromosome 3L. The data represent the cardiac measurements in adult flies obtained by OCT for end-diastolic dimension (EDD) in microns, end-systolic dimension (ESD) in microns, and fractional shortening (FS). FS was calculated as (EDD-ESD)/EDD x 100. Values are expressed as the mean +/- SE. N = 14−16 samples per group. Additionally, the data is represented in a binary manner with “Dilated” defined as an EDD > 90 microns and “Impaired Systolic Function” defined as an ESD > 20 microns) where “N” = no and “Y” = yes. (0.07 MB DOC) [file pgen.1000969.s006.doc]

**Table S1. Cardiac parameters from the initial screen of genomic deficiencies along *Drosophila* Chromosome 3L.**

| **Stock** | **EDD ± SE (microns)** | **ESD ± SE (microns)** | **FS ± SE (%)** | **Dilated**  **(EDD > 90 microns)** | **Impaired systolic function (ESD > 20 microns)** |
| --- | --- | --- | --- | --- | --- |
| ***w^1118^*** | **66 ± 8** | **<10** | **>84** | **N** | **N** |
| ***Df(3L)Exel6083/+*** | **79 ± 5** | **<10** | **>87** | **N** | **N** |
| ***Df(3L)Exel6084/+*** | **74 ± 6** | **<10** | **>86** | **N** | **N** |
| ***Df(3L)Exel6085/+*** | **65 ± 5** | **<10** | **>85** | **N** | **N** |
| ***Df(3L)Exel6086/+*** | **78 ± 5** | **<10** | **>87** | **N** | **N** |
| ***Df(3L)Exel6087/+*** | **77 ± 4** | **<10** | **>87** | **N** | **N** |
| ***Df(3L)Exel6088/+*** | **76 ± 11** | **<10** | **>87** | **N** | **N** |
| ***Df(3L)Exel6089/+*** | **74 ± 6** | **15 ± 3** | **80 ± 4** | **N** | **N** |
| ***Df(3L)Exel6090/+*** | **74 ± 3** | **<10** | **>86** | **N** | **N** |
| ***Df(3L)Exel6091/+*** | **68 ± 6** | **<10** | **>85** | **N** | **N** |
| ***Df(3L)Exel6092/+*** | **81 ± 5** | **<10** | **>88** | **N** | **N** |
| ***Df(3L)Exel6093/+*** | **63 ± 7** | **<10** | **>84** | **N** | **N** |
| ***Df(3L)Exel6094/+*** | **74 ± 5** | **<10** | **>86** | **N** | **N** |
| ***Df(3L)Exel6095/+*** | **89 ± 8** | **32 ± 9** | **63 ± 3** | **N** | **Y** |
| ***Df(3L)Exel6096/+*** | **65 ± 6** | **<10** | **>85** | **N** | **N** |
| ***Df(3L)ED4079/+*** | **80 ± 5** | **<10** | **>88** | **N** | **N** |
| ***Df(3L)ED4177*/+** | **83 ± 6** | **<10** | **>88** | **N** | **N** |
| ***Df(3L)ED4191*/+** | **106 ± 4*** | **43 ± 3*** | **59 ± 3** | **Y** | **Y** |
| ***Df(3L)ED4196*/+** | **121 ± 4*** | **55 ± 5*** | **55 ± 3** | **Y** | **Y** |
| ***Df(3L)ED201/+*** | **76 ± 6** | **<10** | **>87** | **N** | **N** |
| ***Df(3L)ED202*/+** | **75 ± 2** | **<10** | **>87** | **N** | **N** |
| ***Df(3L)ED4238/+*** | **103 ± 4** | **37 ± 4** | **64 ± 2** | **Y** | **Y** |
| ***Df(3L)ED4256/+*** | **86 ± 5** | **24 ± 7** | **75 ± 7** | **N** | **Y** |
| ***Df(3L)ED4284/+*** | **80 ± 7** | **<10** | **>88** | **N** | **N** |
| ***Df(3L)ED4293/+*** | **76 ± 8** | **<10** | **>87** | **N** | **N** |
| ***Df(3L)ED207*/+** | **109 ± 4*** | **49 ± 3*** | **55 ± 3** | **Y** | **Y** |
| ***Df(3L)ED208/+*** | **86 ± 6** | **14 ± 4** | **87 ± 5** | **N** | **N** |
| ***Df(3L)ED210/+*** | **84 ± 8** | **27 ± 6** | **67 ± 6** | **N** | **Y** |
| ***Df(3L)ED4342/+*** | **83 ± 5** | **<10** | **>88** | **N** | **N** |
| ***Df(3L)ED211/+*** | **82 ± 7** | **<10** | **>88** | **N** | **N** |
| ***Df(3L)ED4287/+*** | **76 ± 10** | **16 ± 5** | **80 ± 6** | **N** | **N** |
| ***Df(3L)ED4470/+*** | **77 ± 5** | **<10** | **>87** | **N** | **N** |
| ***Df(3L)ED4288/+*** | **80 ± 7** | **<10** | **>88** | **N** | **N** |
