# Supplementary material for: Affecting Rhomboid-3 Function Causes a Dilated Heart in Adult Drosophila
Source: PLoS Genet. 2010 May 27;6(5):e1000969. doi: 10.1371/journal.pgen.1000969 (PMC2877733; doi:10.1371/journal.pgen.1000969)
Supplement: Table S2 — Expression of rho1 and rho3 mRNA in different developmental stages and adult tissues. Representative quantitative real-time RT-PCR. Total RNA and cDNA were prepared from embryos in indicated time points after birth, larvae, pupae, and dissected adult tissues of w1118. Summary data show relative gene expression of rho3 and rho1 at developmental stages and in adult tissues (expressed as fold-change compared to the rho3 or rho1 expression level at 0–8h embryo, respectively). Data represent mean ± SE of at least three independent experiments, each performed in triplicate. (0.03 MB DOC) [file pgen.1000969.s007.doc]

**Table S2. Expression of *rho1* and *rho3* mRNA in different developmental stages and adult tissues.**

| **Developmental Stage:** | **rho3** | **rho1** |
| --- | --- | --- |
| **Embryo 0-8 hr** | **1.00** | **1.00** |
| **Embryo 8-16 hr** | **21.62 ± 0.27** | **1.56 ± 0.13** |
| **Embyro 16-24 hr** | **10.52 ± 0.56** | **0.86 ± 0.02** |
| **Larvae** | **10.30 ± 1.02** | **0.18 ± 0.01** |
| **Pupae** | **124.91 ± 3.35** | **0.99 ± 0.06** |
|  |  |  |
| **Adult Tissue:** |  |  |
| **Head** | **59.50 ± 5.20** | **2.20 ± 0.26** |
| **Thorax** | **20.10 ± 1.50** | **1.05 ± 0.03** |
| **Abdomen** | **5.10 ± 0.10** | **0.21 ± 0.02** |
| **Heart** | **0.20 ± 0.04** | **0.02 ± 0.01** |
| **Leg** | **59.40 ± 2.10** | **1.77 ± 0.10** |
